# Cyclosporine modulates neutrophil functions via the SIRT6–HIF‐1α–glycolysis axis to alleviate severe ulcerative colitis

**DOI:** 10.1002/ctm2.334

**Published:** 2021-02-14

**Authors:** Huiying Lu, Jian Lin, Chunjin Xu, Mingming Sun, Keqiang Zuo, Xiaoping Zhang, Mingsong Li, Hailiang Huang, Zhong Li, Wei Wu, Baisui Feng, Zhanju Liu

**Affiliations:** ^1^ Center for IBD Research Department of Gastroenterology Shanghai Tenth People's Hospital of Tongji University Shanghai China; ^2^ Department of Gastroenterology First People's Hospital of Shangqiu City Affiliated to Xinxiang Medical University Shangqiu China; ^3^ Department of Gastroenterology Third Affiliated Hospital of Guangzhou Medical University Guangzhou China; ^4^ Analytic and Translational Genetics Unit Massachusetts General Hospital Boston Massachusetts USA; ^5^ Department of Medicine Harvard Medical School Boston Massachusetts USA; ^6^ Broad Institute of Harvard and MIT Cambridge Massachusetts USA; ^7^ Shanghai Cell Therapy Group Shanghai China; ^8^ Department of Gastroenterology Second Affiliated Hospital of Zhengzhou University Zhengzhou China

**Keywords:** glycolysis, neutrophil, SIRT6, ulcerative colitis

## Abstract

**Background:**

Cyclosporine A (CsA) is routinely used to treat patients with steroid‐refractory acute severe ulcerative colitis (ASUC). Here, we studied the underlying mechanisms of CsA‐mediated alleviation in ASUC patients.

**Methods:**

Neutrophil functions including expression of cytokines, apoptosis, and migration were measured by qRT‐PCR, flow cytometry, and Transwell assay. Dynamic changes of glycolysis and tricarboxylic acid (TCA) cycle were measured by a Seahorse extracellular flux analyzer. Gene differences were determined and verified by RNA sequencing, qRT‐PCR, and Western blotting. Small interfering RNA and inhibitors were used to knock down Sirtuin 6 (SIRT6) in HL‐60 cells and block expression of SIRT6, hypoxia‐inducible factor‐1α (HIF‐1α), and pyruvate dehydrogenase lipoamide kinase isozyme 4 (PDK4) in neutrophils.

**Results:**

We found that HIF‐1α expression and glycolysis significantly increased, while the release of IL‐8, myeloperoxidase (MPO) and reactive oxygen species (ROS), the apoptosis, and ability of migration markedly decreased in neutrophils of ASUC patients who responded to CsA (Response group) compared with those who did not respond to CsA (Nonresponse group). We also observed that CsA‐induced functional alternation of neutrophils was initiated through suppressing SIRT6 expression, which is responsible for expression of the downstream signaling molecules (e.g., HIF‐1α, PFKFB3) and PDK4 ubiquitination, leading to fueling neutrophil glycolysis and TCA cycle. Furthermore, blockage of SIRT6 signaling demonstrated to be the same functional changes as CsA to decrease the migration of neutrophils.

**Conclusions:**

The data reveal a novel mechanism of CsA in alleviating ASUC by promoting neutrophil HIF‐1α expression and restricting excessive neutrophil activation in a SIRT6−HIF‐1α−glycolysis axis, suggesting SIRT6 as a candidate target for maintaining mucosal homeostasis and treating intestinal inflammation.

## Graphical abstract

(1) CsA therapy suppresses excessive activation and facilitates HIF‐1α production and glycolysis of neutrophils from the Response group but not the Nonresponse group of ASUC patients. (2) CsA induces clinical remission of ASUC patients through amplifying both the glycolysis and aerobic oxidation in neutrophils through SIRT6–HIF‐1α–glycolysis axis. (3) Blockade of SIRT6 and HIF‐1α regulates neutrophil functions.

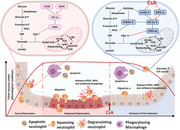

**Abbreviations**
2‐DG: 2‐deoxy‐D‐glucose
Akt: threonine kinase
ASUC: acute severe ulcerative colitis
CARD8: caspase recruitment domain‐containing protein 8
CCL3/MIP‐1‐α: chemokine (C–C motif) ligand 3/macrophage inflammatory protein 1‐alpha
CsA: cyclosporine A
ECAR: extracellular acidification rate
HC: healthy control
HIF‐1α: hypoxia‐inducible factor‐1α
MPO: myeloperoxidase
mTOR: the mechanistic target of rapamycin
NF‐AT: nuclear factor of activated T cells
OCR: oxygen consumption rate
PDK4: pyruvate dehydrogenase lipoamide kinase isozyme 4
PFKFB3: 6‐phosphofructo‐2‐kinase/fructose‐2,6‐biphosphatase 3
ROS: reactive oxygen species
SIRT6: Sirtuin 6
TCA: tricarboxylic acid
UC: ulcerative colitis

## 1 INTRODUCTION

Ulcerative colitis (UC) is generally referred to as a chronic disorder with energy deficiency characterized by continuous mucosal inflammation extending proximally from the rectum.^1^, ^2^ Although the precise etiology and pathology remain unknown, the dysregulated immune responses in inflamed mucosa of UC are mainly characterized by inflammatory infiltration of neutrophils together with other activated immune cells (e.g., T cells, macrophages, and dendritic cells).^1^, ^2^ The uncontrollable inflammation even leads to neutrophilic cryptitis, crypt abscesses, and mucosal ulceration.^3^ Increasing lines of evidence have demonstrated that tissue homeostasis, damage and repair in gut mucosa depend on hypoxia inducible factor‐1α (HIF‐1α), a modulator of glucose consumption,^4^, ^5^, ^6^ which is increased during colitis by a variety of stimuli such as LPS^7^ and TNF‐α^8^ that is involved in the NF‐κB‐dependent pathway, and subsequently regulates mucosal homeostasis by facilitating the expression of adrenomedullin^7^ or IL‐33.^5^, ^6^

Cyclosporine A (CsA), one of calcineurin inhibitors, is routinely utilized as an inducer for clinical remission in severe steroid‐refractory UC,^9^ which has been demonstrated to inhibit the dephosphorylation of nuclear factor of activated T cells (NF‐AT) by interacting with calcineurin and subsequently translocating to the nucleus.^10^ However, the underlying mechanisms whereby CsA modulates immune responses in gut mucosa are still elusive. Given that calcineurin inhibitor could to some extent modulate neutrophil functions^11^ and that granulocyte colony‐stimulating factor, an inducer of neutrophil accumulation, leads to the remission of acute colitis,^12^, ^13^ we speculate that neutrophils may be actually associated with the efficacy of CsA in the management of severe UC.

Neutrophils have long been considered as the short‐lived effector cells, act as double‐edged swords in acute inflammatory response^14^ and play an important role in maintaining intestinal homeostasis and mucosal healing at the early stage of acute severe ulcerative colitis (ASUC).^15^, ^16^, ^17^ Under physiological conditions, the metabolism of glucose in neutrophils is mainly based on glycolysis,^5^ which is highly regulated by HIF‐1α,^18^ and neutrophils thus play an essential role in different pathophysiological processes including chronic intestinal inflammation and mucosal healing.^19^ When triggered by microbial or inflammatory stimuli, activated neutrophils could migrate and accumulate at the site of infected or injured intestinal mucosa, recognize stimuli and undergo phagocytosis, degranulation, release of reactive oxygen species (ROS), myeloperoxidase (MPO), as well as the formation of neutrophil extracellular traps to eradicate intestinal pathogens and thus promoting the alleviation of intestinal mucosal inflammation in patients with ASUC.^16^, ^20^, ^21^, ^22^ In addition, chemokines or cytokines produced by neutrophils are also involved in the orchestration of immune responses of gut mucosa as well as the functions of immune cells.^23^ Furthermore, apoptotic neutrophils are recognized and cleared by macrophages in the inflamed intestinal mucosa to maintain their own homeostasis.^21^, ^24^ However, excessive accumulation and activation of neutrophils in colonic crypts during the advanced phases of ASUC give rise to cryptitis and ultimately to crypt abscess concomitantly hindering intestinal mucosal restoration.^25^ Therefore, programmed balancing neutrophil immune regulation between anti‐inflammatory protective functions (e.g., degranulation, phagocytosis, bactericidal activity) and detrimental effects on intestinal tissue (e.g., cryptitis, crypt abscess, ulceration) play an indispensable role in the initiation and limitation of mucosal inflammation in ASUC.^26^, ^27^

In the present study, we investigated the functional alterations of neutrophils from patients with ASUC who responded to CsA therapy and found that neutrophils had a decrease of the migration, apoptosis, and MPO and ROS production but an increase of HIF‐1α expression and glycolysis. RNA sequencing and metabolic analysis revealed that CsA facilitated neutrophil glycolysis and tricarboxylic acid (TCA) cycle via the suppression of Sirtuin 6 (SIRT6), an upstream gene of HIF‐1α, and pyruvate dehydrogenase lipoamide kinase isozyme 4 (PDK4), a downstream gene of HIF‐1α. Consequently, blockage of the SIRT6 signaling resulted in the same functional changes of neutrophils as CsA did. Therefore, our results indicate that CsA alleviates intestinal mucosal damage by directly modulating neutrophil functions through the SIRT6−HIF‐1α−glycolysis axis and inducing clinical remission in severe UC.

## 2 MATERIALS AND METHODS

### 2.1 Patients

All patients recruited in this study were diagnosed for ASUC according to a Lichtiger score^28^ >10 points and a Mayo score^29^ ≥11 based on a combination of symptoms, endoscopic findings, histology and biomarkers of inflammation, who were not receiving biologics (e.g., infliximab, adalimumab, and vedolizumab) or calcineurin inhibitors (e.g., CsA, tacrolimus) before the study and had no relief after the treatment of glucocorticoids for 5 days.^30^ Patients with a history of colorectal dysplasia, colorectal polyps, colorectal cancer, Crohn's disease or indeterminant colitis; a positive test of hepatitis B or C virus infection, or active bacterial and/or microorganism infections were excluded. All patients were recruited from three university hospitals: the Shanghai Tenth People's Hospital of Tongji University, the First People's Hospital of Shangqiu City Affiliated to Xinxiang Medical University, and the Second Affiliated Hospital of Zhengzhou University between October 2016 and June 2020. Fifty‐three steroid‐refractory patients with ASUC received CsA therapy (Ciclosporin Injection, Novartis Pharma Schweiz AG, Rotkreuz, Switherland) at a dosage of 3 mg/kg/day for 7 days,^31^ and the details of the clinical information were displayed in Table 1. The doses were adjusted based on blood concentrations from 250 to 300 ng/ml by HPLC/MS. Patients who achieved clinical response after 7 days of iv injection (defined by Lichtiger score ≤10 with decrease of at least 3 points) then received oral administration of CsA (4−5 mg/kg; Ciclosporin Capsules, Novartis Pharma Schweiz AG) for 3 months, and mucosal healing 3 months after oral administration of CsA was assessed by Mayo disease activity index endoscopic subscore of 0–3.^28^, ^31^ We obtained blood samples from these patients before and on the eighth day of treatment, isolated neutrophils immediately, and named these samples as day 0 (D0) and day 8 (D8), respectively (Figure S1). Detailed clinical information of patients who met the inclusion criteria but did not receive CsA therapy is displayed in Table S1. Peripheral blood samples from healthy volunteers were also harvested and used as controls. EDTA‐anticoagulated blood samples (20 ml) were obtained from UC patients and healthy donors/controls (HC). This study was approved by the Ethics Committee of the Shanghai Tenth People's Hospital of Tongji University (SHSY‐IEC‐4.0/19‐52/01). Written informed consent was obtained from each subject.

**Highlights**

1. CsA therapy suppresses the apoptosis, migration, the release of antibacterial peptides, ROS and MPO, and facilitates HIF‐1α production and glycolysis of neutrophils in the Response group but not in the Nonresponse group of ASUC patients.
2. CsA induces clinical remission in ASUC patients by amplifying both glycolysis and aerobic oxidation in neutrophils through the SIRT6−HIF‐1α−glycolysis axis independent of functional suppression of T cells.
3. Differential genes of neutrophils involved in inducing clinical remission in ASUC patients are associated with apoptotic process, metabolic process, immune response, cytoskeleton, protein polyubiquitination, and autophagy.
4. Blockade of SIRT6 and HIF‐1α regulates the apoptosis and migration of neutrophils on the treatment with CsA.

**TABLE 1:** The changes of clinical characteristics of ulcerative colitis (UC) patients who received cyclosporine A (CsA) treatment

							Lichtiger score		Mayo score		PMN (× 10^9^)		Proportion of PMN (%)	
Patient number	Sex	Age (years)	Duration of disease (months)	Lesion range^a^	Side effects	Curative effect	Before	Day8	Before	Day98	Before	Day8	Before	Day8
ASUC01	F	47	12	E3	/	Response	10	1	11	2	6.32	2.32	79.7	42.2
ASUC02	F	25	48	E3	/	Failure	13	10	12	8	7.81	8.3	78.2	80.0
ASUC03	F	65	168	E2	/	Response	10	0	12	3	4.37	2.43	64.7	57.5
ASUC04	F	48	1	E2	/	Response	10	0	11	1	6.32	3.06	61.1	47.6
ASUC05	M	61	16	E1	/	Failure	16	11	11	11	3.91	4.13	70.9	69.8
ASUC06	F	51	6	E2	/	Response	14	2	11	3	5.23	3.46	60.5	50.1
ASUC07	M	37	120	E3	/	Response	10	0	11	1	9.4	5.01	79.6	59.7
ASUC08	M	35	36	E2	/	Response	10	1	12	5	3.63	2.56	63.5	57.6
ASUC09	M	20	6	E2	/	Response	12	0	11	0	11.13	4.42	79.9	60.2
ASUC10	M	65	96	E2	Fever of unknown origin	Response	13	1	11	2	5.77	2.91	80.8	66.0
ASUC11	F	64	108	E3	Hepatic event	Response	15	2	11	2	12.78	4.27	81.7	67.8
ASUC12	F	33	24	E3	Renal event	Failure	13	12	11	10	6.91	4.87	70	56.6
ASUC13	F	21	8	E3	/	Response	17	2	11	0	5.78	3.92	78.4	65.9
ASUC14	F	21	1	E3	/	Response	11	1	11	0	13.86	5.38	86.3	65.9
ASUC15	M	61	36	E3	/	Failure	14	10	12	12	4.79	2.97	58.8	51.8
ASUC16	M	30	60	E3	Paresthesias	Failure	11	8	12	10	7.58	5.07	77.7	69.8
ASUC17	M	27	60	E3	/	Response	12	3	11	2	7.13	4.25	71.8	62.5
ASUC18	F	36	11	E2	/	Response	11	4	12	6	8.14	3.17	85.4	60.4
ASUC19	M	29	2	E3	Fever of unknown origin	Response	14	4	12	3	11.30	6.94	89.8	75.9
ASUC20	M	45	72	E3	/	Response	17	3	12	1	8.32	3.11	80.2	62.5
ASUC21	M	62	60	E3	/	Response	14	2	12	2	7.38	2.7	76.2	53.4
ASUC22	F	53	36	E3	Renal event	Failure	11	9	11	9	10.35	8.91	87.4	86.4
ASUC23	M	30	9	E3	Hepatic event	Failure	13	12	12	11	6.56	7.51	79.2	78.4
ASUC24	M	28	12	E2	/	Response	13	3	11	3	7.25	2.55	63.9	41.8
ASUC25	F	32	1	E3	/	Response	13	5	12	7	6.92	2.46	70.8	51.5
ASUC26	F	63	120	E3	Hepatic event	Failure	12	11	11	10	7.74	6.32	73.9	69.3
ASUC27	F	36	24	E3	Renal event	Failure	15	15	12	12	7.41	6.89	83.4	80.4
ASUC28	F	29	10	E3	/	Response	10	3	11	4	8.19	3.27	79.7	57.4
ASUC29	M	60	48	E3	/	Response	12	4	12	6	10.77	3.7	86.4	53.2
ASUC30	F	39	6	E3	/	Response	13	2	12	2	10.8	2.15	77.4	60.4
ASUC31	M	48	48	E3	Hepatic event	Failure	12	12	12	11	8.59	8.93	81.2	86.4
ASUC32	M	35	52	E3		Response	16	6	12	3	7.41	3.15	80.3	65.8
ASUC33	M	44	40	E3		Response	13	4	11	4	9.10	3.05	81.2	70.6
ASUC34	F	39	24	E3		Response	14	4	11	3	7.52	3.55	77.8	60.1
ASUC35	M	57	78	E3		Failure	15	12	12	11	10.88	12.22	70.3	72.8
ASUC36	M	61	90	E3		Response	13	2	12	3	6.02	3.40	73.1	62.5
ASUC37	F	31	4	E3		Failure	17	12	12	10	8.61	9.22	83.9	79.6
ASUC38	F	38	30	E2		Failure	13	10	12	9	8.48	8.71	68.5	69.3
ASUC39	M	52	108	E3	Hepatic event	Failure	13	12	12	11	10.06	8.35	75.7	70.2
ASUC40	F	60	96	E2		Response	13	6	12	7	9.05	3.01	82.8	61.3
ASUC41	M	47	6	E3		Response	14	3	12	3	9.71	3.09	70.3	59.4
ASUC42	M	49	12	E3		Response	12	3	11	2	7.95	2.61	69.3	60.4
ASUC43	F	23	2	E3		Response	13	2	12	2	10.40	3.27	83.7	70.1
ASUC44	F	44	42	E3	Paresthesias	Failure	16	13	12	9	8.59	7.87	80.4	76.6
ASUC45	F	55	124	E3		Response	13	5	11	5	8.30	4.96	77.6	65.9
ASUC46	M	43	30	E3		Response	13	2	11	1	10.89	3.59	79.5	53.8
ASUC47	F	37	18	E3		Response	12	2	12	3	9.35	3.15	83.8	50.7
ASUC48	F	46	48	E3	Paresthesias	Failure	13	11	12	10	8.07	8.81	69.8	72.5
ASUC49	M	42	36	E3		Response	13	4	11	1	9.73	3.71	75.4	69.1
ASUC50	M	49	24	E2		Response	13	5	12	3	10.46	3.08	74.8	67.2
ASUC51	F	21	15	E3		Response	13	4	12	2	6.45	3.04	68.8	45.9
ASUC52	F	57	36	E3		Response	13	6	12	7	8.84	3.24	73.3	49.5
ASUC53	M	28	12	E3		Failure	14	11	12	11	8.44	6.06	78.3	72.6

^a^ According to the Montreal classification.

### 2.2 Isolation and incubation of peripheral blood neutrophils

Peripheral blood neutrophils, typically present at high density in the circulation, from healthy volunteers and ASUC patients were isolated from the sediment with the red blood cell fraction in a Ficoll‐density gradient.^21^, ^23^ After discarding the supernatants, the neutrophils and erythrocytes were treated with lysing buffer twice to eliminate erythrocytes, and neutrophils were resuspended in PBS before incubation. Isolated neutrophils (5 × 10^6^ cells/ml) were resuspended in RPMI‐1640 medium supplemented with 0.5% fetal bovine serum (FBS) and 1% penicillin/streptomycin (P/S) before treatment. Incubations were performed at 37°C in the presence of 5% CO2.

### 2.3 Isolation and incubation of CD4^+^ T cells

Peripheral blood mononuclear cells were isolated from healthy volunteers or patients with ASUC over Ficoll density gradients, and CD4^+^ T cells were then purified using antihuman CD4 particles (BD Biosciences; San Diego, CA, USA). These CD4^+^ T cells were counted and seeded in an anti‐CD3 mAb (5 μg/ml)‐coated 24‐well plate (1 × 10^6^ cells/ml for qRT‐PCR, and 5 × 10^5^ cells/ml for proliferation assay) in the presence of anti‐CD28 mAb (2 μg/ml) in RPMI‐1640 medium supplemented with 10% FBS, 1% P/S, sodium pyruvate (1 mM; Life Technologies; Carlsbad, CA, USA), and β‐mercaptoethanol (50 μM; Sigma‐Aldrich).^32^, ^33^ We cultured CD4^+^ T cells in the absence or presence of CsA (10 μg/ml) and harvested cells for qRT‐PCR (cultured for 48 h) and proliferation assay (cultured for 5 days), respectively.

### 2.4 Assessment of neutrophil apoptosis

For analyzing the apoptosis of neutrophils after treatment with LPS or CsA for 3 h, an Annexin V‐fluorescein isothiocyanate (FITC) Apoptosis Detection Kit (ebioscience; San Diego, CA, USA) was used according to the manufacturer's instructions. Briefly, neutrophils isolated from peripheral blood were incubated for 3 h without any treatment, or exposed to LPS (300 ng/ml), CsA (10 μg/ml), and LPS (300 ng/ml) together with CsA (10 μg/ml), respectively, and then resuspended in binding buffer at a concentration of 1 × 10^6^ cells/ml. Subsequently, cells were stained with FITC‐conjugated annexin V and PI for 15 min at room temperature.^34^ Flow cytometric analysis was performed on a BD FACSCanto II (BD Biosciences; San Diego, CA, USA) and analyzed by FlowJoVX software (Tree Star, Inc.; Ashland, OR, USA).

### 2.5 Transwell migration assay for neutrophils

Isolated neutrophils were resuspended in serum‐free RPMI‐1640 medium containing 1% P/S at 10^6^/ml. Thereafter, 200 μl of cells were seeded in the upper well of a Transwell chamber in the absence or presence of CsA (10 μg/ml) with microporous filters (3‐μm pores, Millpore; Billerica, MA, USA), and 500 μl of 1% P/S serum‐free RPMI‐1640 medium in the absence or presence of *N*‐formylmethionyl‐leucyl‐phenyl‐alanine (fMLP) (50 nM) was added into the bottom chamber. After incubation for 3 h, cells adherent on the membrane in 3‐μm pores Transwell system were fixed with 4% paraformaldehyde (PFA) for 10 min, stained with 1% crystal violet for 30 min, and then observed under a light microscope (×200).^35^, ^36^ Thereafter, we performed the decolorization of crystal violet via acetic acid and counted the cells by measuring the OD values. Nondecolorized acetic acid was used as medium alone to reduce the impact of background.

### 2.6 Transmission electron microscopy (TEM)

Neutrophils isolated from peripheral blood were incubated with or without CsA (10 μg/ml) for 3 h. These cells were flooded with EM grade glutaraldehyde fixative buffer containing 2% glutaraldehyde, 2% paraformaldehyde, and 0.1 M cacodylate, and stored at 4˚C before the processing of electron microscopy. The images were taken by Hitachi TEM system (Tokyo, Japan). Quantification of mitochondria per cell was performed using Image J. software by two TEM specialists blind to the experiments.

### 2.7 Seahorse extracellular flux analyzer assays for glycolysis

Neutrophils were isolated and seeded directly into Cell‐Tak‐Coated Seahorse XF96 cell culture microplates (Agilent Technologies). The extracellular acidification rate (ECAR) was measured using a Seahorse XFe96 Analyzer (Agilent Technologies). Sensor cartridges were prehydrated in XF calibrant solution overnight in a CO_2_‐free incubator and then loaded with glucose (10 mM, Port A), oligomycin (2 μM, Port B), and 2‐deoxyglucose (50 mM, Port C), respectively, after injection. Data were analyzed using the XF Glycolysis Stress Test Report Generator (Agilent Technologies).

### 2.8 Seahorse extracellular flux analyzer assays for oxidative phosphorylation (OXPHOS)

The assay was performed as described above, except the XF assay medium supplemented with 2‐mM glutamine, 1‐mM sodium pyruvate, and 10‐mM glucose. The sensor cartridge was loaded with oligomycin (10 μM, Port A), FCCP (1 μM, Port B), and rotenone/antimycin A (0.5 μM, Port C), respectively, after injection. Data were analyzed using the XF Mito Stress Test Report Generator.

### 2.9 Statistics analysis

Statistical comparisons were performed using Tukey's test or two‐tailed Student's *t*‐test, as indicated. All data were expressed as mean ±  SEM and analyzed using SPSS V.20.0 (SPSS; Chicago, IL, USA). GraphPad Prism statistical software (version 6) was used to plot figures. *p*‐Value < .05 was considered to be statistically significant (Please refer to Supporting Materials and Methods for other information).

## 3 RESULTS

### 3.1 CsA therapy decreases neutrophil infiltration in inflamed colon of ASUC patients in the Response group but not in the Nonresponse group

Fifty‐three patients with ASUC who were steroid‐refractory and eligible for the alternative medical therapy with CsA during the study period were recruited as described in the Methods section. Initially, they received treatment with intravenous injection of CsA for 7 days as an induction therapy. Of all these patients, 36 were classified as the Response group as defined by Lichtiger score ≤10 with decrease of at least 3 points, while the remaining 17 patients were classified as the Nonresponse group (Table 1).^28^, ^31^ Of 17 patients who were not responding to CsA treatment, 12 patients underwent colectomy and five patients received and responded to infliximab treatment. All patients in the Response group received oral administration of CsA as maintenance therapy for 3 months after 7‐day induction therapy with iv injection of CsA (Figure S1).

**FIGURE 1:**
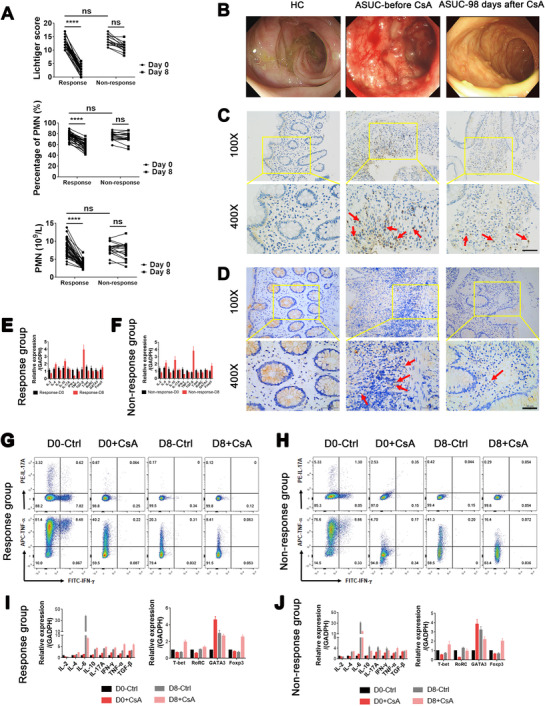
Cyclosporine A (CsA) induces clinical remission and mucosal healing and decreases leukocyte infiltration in inflamed colon of acute severe ulcerative colitis (ASUC) patients from the Response group. (A) Patients with ASUC received treatment with CsA at a dose of 3 mg/kg/day for 7 days, and clinical parameters including Lichtiger score, polymorphonuclear neutrophil (PMN) count, and percentage of PMN in these patients of the Response group (*n* = 36) and the Nonresponse group (*n* = 17) are shown. (B–D), Patients in the Response group (*n* = 36) underwent colonoscopy before treatment (day 0) and 3 months (day 98) after maintenance treatment with oral administration of CsA, and the colonic biopsies were collected for immunohistochemical staining. (B) Representative images of colonoscopy showing colonic mucosal appearance in the ascending colon from a healthy donor (HC) and changes of intestinal mucosal lesions from an ASUC patient of the Response group before (day 0, middle panel) and 3 months (day 98, right panel) after CsA treatment. (C) Representative images of immunohistochemical staining for MPO expression, indicative of neutrophils in inflamed colon from an ASUC patient of the Response group before (day 0, middle panels) and 3 months (day 98, right panels) after CsA treatment and normal colon mucosa from HC (left panels). The arrows indicate neutrophils. Scale bar represents 50 μm. (D) Representative images of immunohistochemical staining for CD4 expression, indicative of CD4^+^ T cells in inflamed colon from ASUC patient of the Response group before (day 0, middle panels) and 3 months (day 98, right panels) after CsA treatment and normal colon mucosa from a HC (left panels). The arrows indicate CD4^+^ T cells. Scale bar represents 50 μm. (E and F) Colon biopsies were collected from ASUC patients from the Response group (E; *n* = 10) and the Nonresponse group (F; *n* = 8) before (D0) and on day 8 (D8) of treatment with CsA, and the mRNA expression of cytokines (e.g., IL‐2, IL‐4, IL‐6, IL‐10, IL‐17A, TNF‐α, TGF‐β, and IFN‐γ) and transcription factors (e.g., T‐bet, RORC, GATA3, and Foxp3) were detected by qRT‐PCR and normalized to GAPDH. (G and H) CD4^+^ T cells (1 × 10^6^ cells) were isolated from ASUC patients before (D0) and on day 8 (D8) of treatment with CsA from the Response group (G; *n* = 36) and the Nonresponse group (H; *n* = 17), seeded in anti‐CD3 mAb (5 μg/ml)‐coated 24‐well plates, and stimulated with anti‐CD28 mAb (2 μg/ml) in the absence (Ctrl) or presence of CsA (10 μg/ml) for 5 days. Intracellular expression of IL‐17A, TNF‐α, and IFN‐γ was stained by fluorochrome‐conjugated antibodies and representative images of flow cytometry were shown. (I and J) CD4^+^ T cells from the Response group (I; *n* = 36) and the Nonresponse group (J; *n* = 17) were cultured and stimulated with immobilized anti‐CD3 mAb and anti‐CD28 mAb as indicated above in the absence (Ctrl) or presence of CsA (10 μg/ml) for 48 h. Cells were harvested, and the mRNA expression of cytokines (e.g., IL‐2, IL‐4, IL‐6, IL‐10, IL‐17A, TNF‐α, TGF‐β, and IFN‐γ) and transcription factors (e.g., T‐bet, RORC, GATA3, and Foxp3) were detected by qRT‐PCR and normalized to GAPDH. Statistical significance was assessed by Tukey's test and two‐tailed Student's *t*‐tests. ****p < .0001

We observed no differences between the Response group and the Nonresponse group before CsA treatment in the numbers and percentages of neutrophils, Lichtiger score, the total numbers of WBC, Mayo score, the levels of C‐reactive protein (CRP), and fecal calprotectin (FC), while they were markedly decreased in the Response group compared with those in the Nonresponse group (Figure 1A, Figure S2A) on day 8 after CsA infusion. Moreover, colonoscopic examination was performed and showed that severe ulcerations were present in inflamed colon before CsA treatment, which were significantly relieved with mucosal healing after a 3‐month maintenance treatment with oral administration of CsA in the Response group (Figure 1B). Immunohistochemical staining revealed that the infiltration of neutrophils (positivity for MPO) and CD4^+^ T cells was remitted in inflamed mucosa after 3‐month treatment of oral administration of CsA (Figure 1C,D). Additionally, the capacities of T‐cell activation and proliferation were markedly inhibited after CsA treatment in both the Response and Nonresponse groups (Figure 1E–J). Thus, we speculated that clinical remission in ASUC patients (36/53, 67.92%) might be mediated by CsA through regulating neutrophil homeostasis independent of functional suppression of T cells.

### 3.2 CsA inhibits the apoptosis and migration of neutrophils from ASUC patients in response to CsA therapy

**FIGURE 2:**
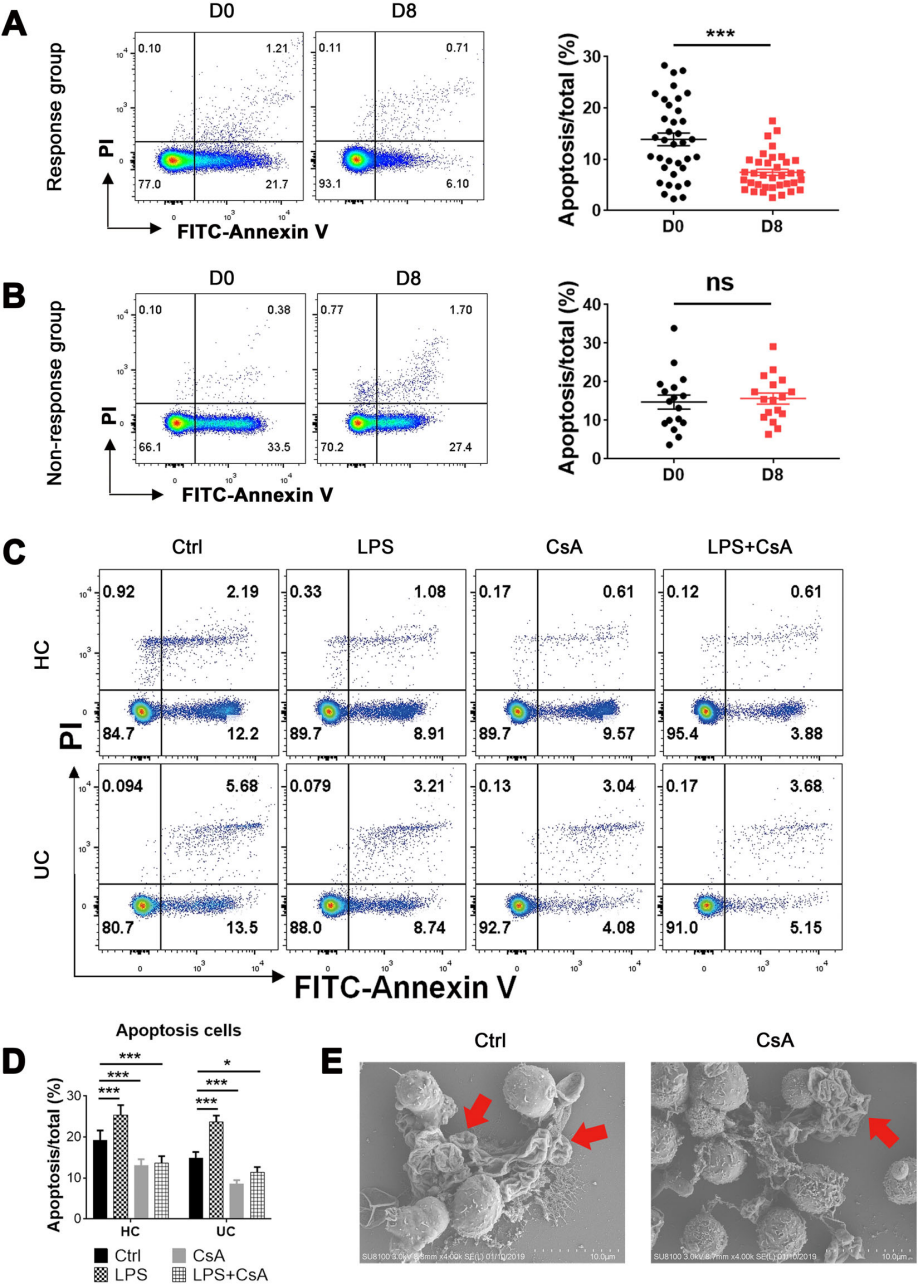
Cyclosporine A (CsA) inhibits the apoptosis of neutrophils in the Response group. (A and B) Neutrophils (1 × 10^6^ cells) were isolated from acute severe ulcerative colitis (ASUC) patients of the Response group (A; *n* = 36) and the Nonresponse group (B; *n* = 17) before (D0) and day 8 (D8) after intravenous injection of CsA. Neutrophils were stained with annexin V, and the representative images of flow cytometry showed the frequencies of apoptotic neutrophils. The percentages of apoptotic neutrophils were shown in right panels. (C and D) Neutrophils (1 × 10^6^ cells) were isolated from peripheral blood of healthy donors (*n* = 25) and naïve patients with ASUC (*n* = 27), pretreated with or without CsA (10 μg/ml) or LPS (300 ng/ml) for 3 h, and then stained with annexin V. (C) Representative images of flow cytometry showed the frequencies of apoptotic neutrophils. (D) The percentages of apoptotic neutrophils were statistically analyzed according to the data from (C). (E) Neutrophils (1 × 10^6^ cells) isolated from naïve ASUC patients were pretreated in vitro in the absence (Ctrl) or presence of CsA (10 μg/ml) for 3 h and then fixed for scanning electron microscopy inspection. Representative scanning electron micrographs of apoptotic cells (red arrows) under magnification ×4.0k (scale bars: 10 μm). Statistical significance was assessed by two‐tailed Student's *t*‐tests and Tukey's test; *p < .05, **p < .01, and ***p < .001; ns, not significant

To determine the potential roles of CsA in regulating the functional and phenotypic changes of neutrophils, we analyzed the apoptosis, migration, and cytokine release of neutrophils from ASUC patients treated with CsA (Table 1). Flow cytometric analysis revealed that CsA significantly inhibited the apoptosis of neutrophils on D8 after CsA therapy in patients of the Response group (Figure 2A) compared with that in the Nonresponse group (Figure 2B). Concurrently, peripheral neutrophils were isolated from ASUC patients and HC and treated with or without CsA (10 μg/ml) or stimulated with LPS (300 ng/ml) in vitro for 3 h to determine the functional changes of neutrophils (Table S1). CsA directly inhibited the apoptosis of neutrophils in vitro in the majority of ASUC patients and healthy controls (Figure 2C,D), which was further confirmed by scanning electron microscopy (SEM) as manifested by a decrease in both cell size and volume (Figure 2E).

**FIGURE 3:**
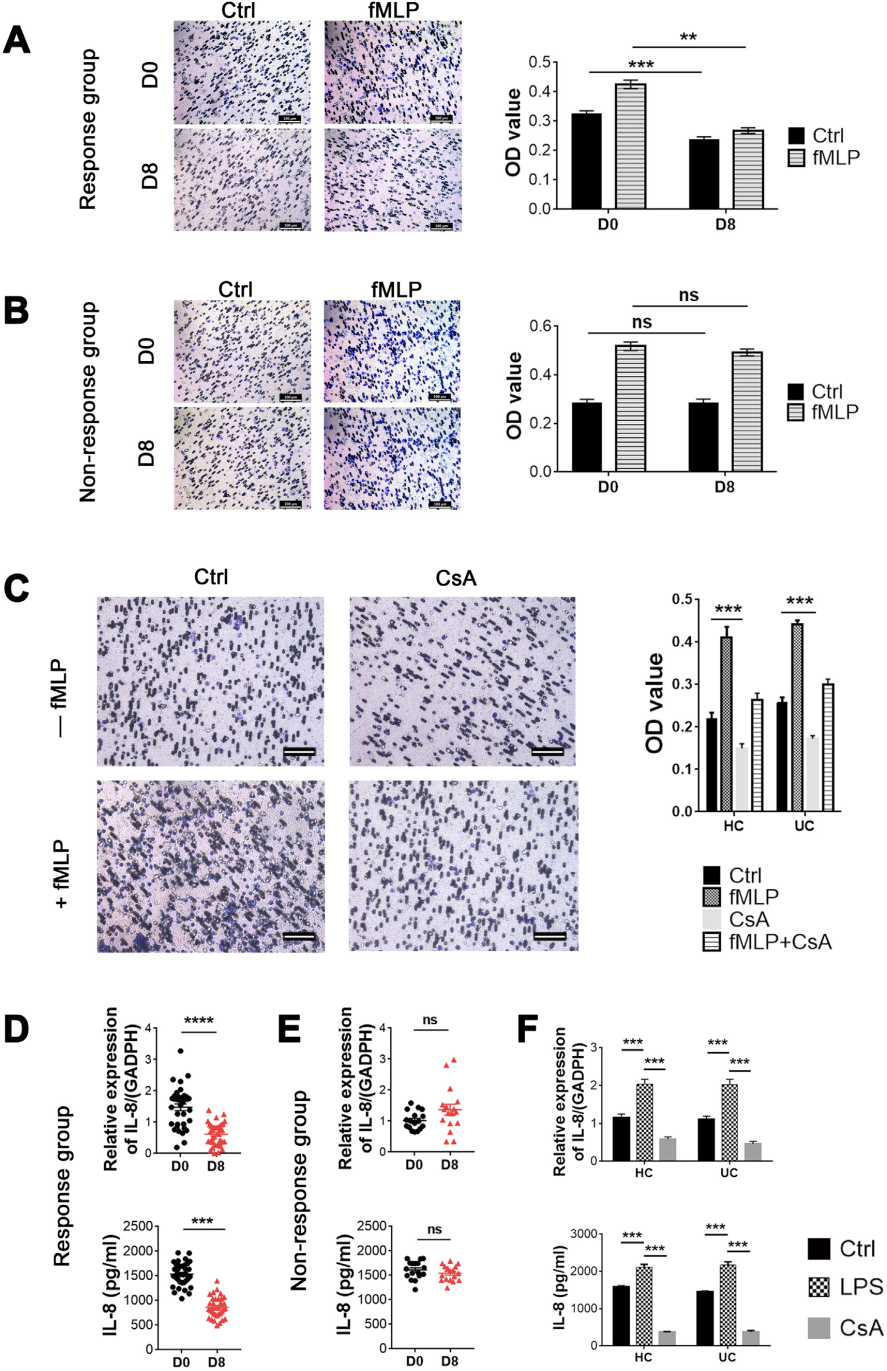
Cyclosporine A (CsA) inhibits the migration and IL‐8 production of neutrophils in the Response group. (A and B) Neutrophils (1 × 10^6^ cells) were isolated from acute severe ulcerative colitis (ASUC) patients of the Response group (A; *n* = 36) and the Nonresponse group (B; *n* = 17) before (D0) and day 8 (D8) after intravenous injection of CsA, and then loaded in the upper chamber of the Transwell chamber. 500 μl of serum‐free RPMI‐1640 medium containing 1% P/S together with or without fMLP (50 nM) was added into the bottom chamber. Scale bars: 100 μm. The migration of neutrophils was quantified by staining with crystal violet and decolorizing with acetic acid, which is shown in the right panel. Nondecolorized acetic acid was used as medium alone to reduce the impact of background. (C) Neutrophils (1 × 10^6^ cells) were isolated from peripheral blood of healthy donors (*n* = 25) and naïve patients with ASUC (*n* = 27), pretreated with or without CsA (10 μg/ml), and loaded in the upper chamber of the Transwell chamber. 500 μl of serum‐free RPMI‐1640 medium containing 1% P/S together with or without (−) fMLP (50 nM) was loaded into the lower chamber. The migration of neutrophils was detected as described in (A). (D and E) Neutrophils (5 × 10^6^ cells) were isolated from the Response group (D; *n* = 36) and the Nonresponse group (E; *n* = 17) as described in (A,B), and the expression of IL‐8 in cultured neutrophils and supernatants were analyzed by qRT‐PCR and ELISA, respectively. (F) Neutrophils (5 × 10^6^ cells) isolated from peripheral blood of healthy donors (*n* = 25) and naïve patients with ASUC (*n* = 27), as indicated in (C), were cultured under stimulation with CsA (10 μg/ml) and LPS (300 ng/ml), respectively, for 3 h. The expression of IL‐8 in cultured neutrophils and supernatants was analyzed by qRT‐PCR and ELISA, respectively. Statistical significance was assessed by two‐tailed Student's *t*‐tests and Tukey's test; *p < .05, **p < .01, ***p < .001, and ****p < .0001; ns, not significant

As for the ability of cell migration, Transwell assay revealed that CsA significantly inhibited the migration of neutrophils on D8 after CsA therapy in patients from the Response group (Figure 3A) compared with that from the Nonresponse group (Figure 3B). In vitro experiments also demonstrated that CsA directly inhibited the migration of neutrophils (Figure 3C) in the majority of ASUC patients and healthy controls. In addition, the inhibition of CsA‐primed neutrophil migration was verified to be significantly suppressed by the downregulation of IL‐8 expression on day 8 after CsA treatment in ASUC patients from the Response group (Figure 3D) compared with that from the Nonresponse group (Figure 3E). IL‐8 expression in neutrophils of ASUC patients and healthy controls was suppressed consistently when stimulated in vitro with CsA (Figure 3F). However, the production of other inflammatory cytokines (e.g., IL‐1β, IL‐6, IL‐10, IL‐12, IL‐17A, TNF‐α, TGF‐β, and IFN‐γ) was not altered in both the Response (Figure S3A) and Nonresponse groups (Figure S3B). No differences were found in these inflammatory cytokines as well (e.g., IL‐1β, IL‐6, IL‐10, IL‐12, IL‐17A, TNF‐α, TGF‐β, and IFN‐γ) between CsA‐treated and untreated neutrophils (Figure S4A,B). Taken together, these data indicate that CsA could inhibit the functions of neutrophils including the apoptosis, migration, and expression of IL‐8 in ASUC patients from the Response group but not from the Nonresponse group.

### 3.3 CsA facilitates expression of HIF‐1α and glycolysis but suppresses the release of ROS, MPO, and antibacterial peptides in neutrophils of ASUC patients in response to CsA therapy

**FIGURE 4:**
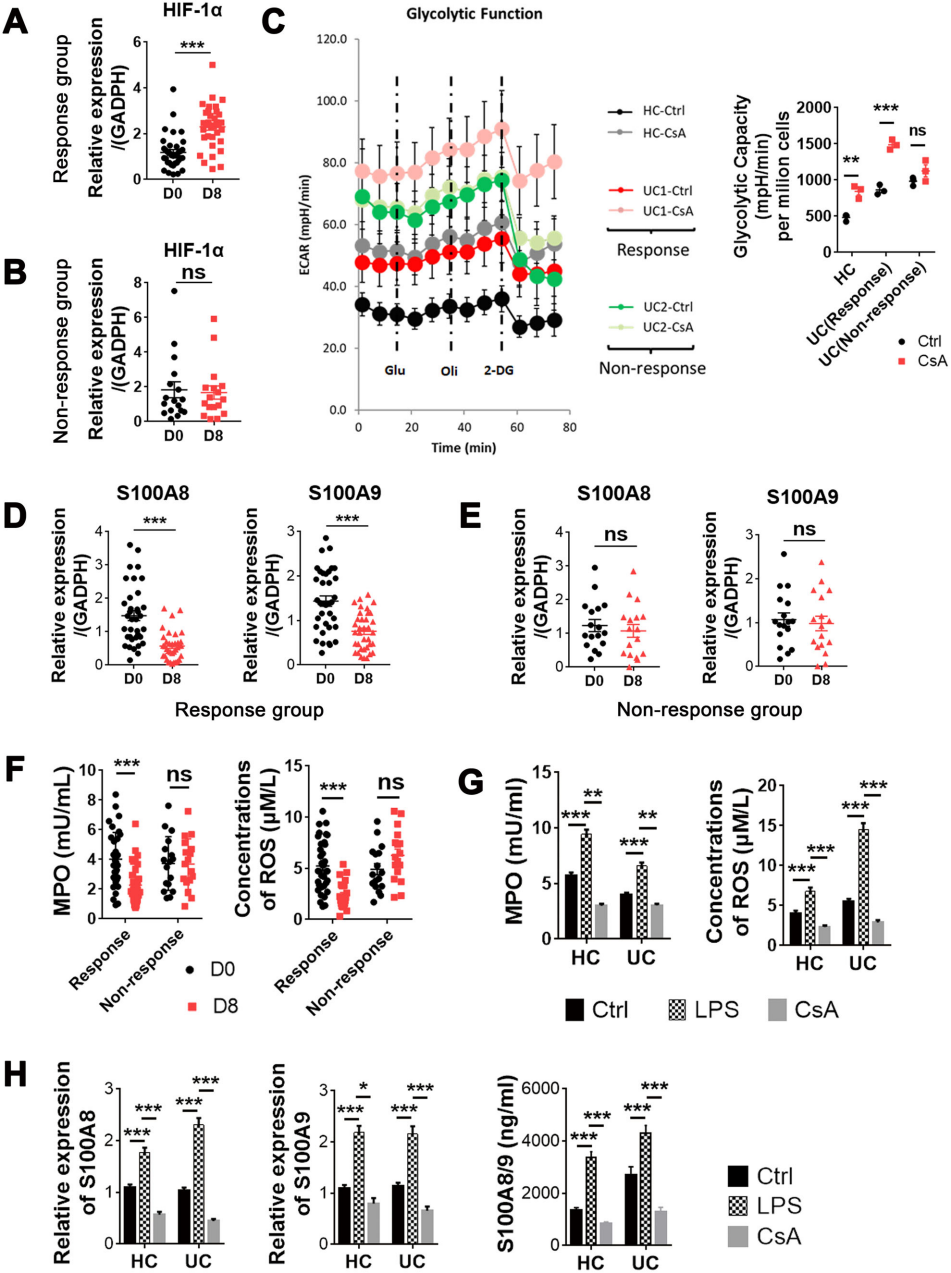
Cyclosporine A (CsA) facilitates neutrophil HIF‐1α expression and glycolysis but inhibits the production of MPO, ROS, and antibacterial peptides in the Response group. (A and B) Neutrophils (5 × 10^6^ cells) were isolated from acute severe ulcerative colitis (ASUC) patients of the Response group (A; *n* = 36) and the Nonresponse group (B; *n* = 17) before (D0) and day 8 (D8) after intravenous injection of CsA, and the mRNA expression of HIF‐1α was detected by qRT‐PCR. (C) Neutrophils (1 × 10^6^ cells) were isolated from healthy donors and ASUC patients of the Response group and the Nonresponse group, and pretreated with or without CsA (10 μg/ml) for 2 h. The dynamic changes of glycolysis in neutrophils were measured by ECAR using a Seahorse extracellular flux analyzer. (D and E) Neutrophils (5 × 10^6^ cells) were isolated, as indicated in (A,B), and the expression of S100A8 and S100A9 in neutrophils was analyzed by qRT‐PCR. (F) Neutrophils (1 × 10^6^ cells) were isolated, as indicated in (A,B), and the levels of MPO and ROS were measured by Amplex Red Hydrogen Peroxide Assay Kit. (G) Neutrophils (1 × 10^6^ cells) were isolated from healthy donors (*n* = 25) and naïve ASUC patients (*n* = 27), and pretreated with or without CsA (10 μg/ml) for 3 h. The levels of MPO and ROS were measured by Amplex Red Hydrogen Peroxide Assay Kit. (H) Neutrophils (5 × 10^6^ cells) were isolated and cultured, as indicated in (G), and the expression of S100A8 and S100A9 was analyzed by qRT‐PCR and ELISA, respectively. Statistical significance was assessed by two‐tailed Student's *t*‐tests and Tukey's test; *p < .05, **p < .01, ***p < .001, and ****p < .0001; ns, not significant

Since HIF‐1α plays an important role in maintaining intestinal homeostasis and glycolysis,^6^, ^37^ we further assessed mRNA expression of HIF‐1α and dynamic changes of glycolysis in neutrophils of ASUC patients treated with CsA. Surprisingly, we found that CsA markedly induced the mRNA expression of HIF‐1α in neutrophils on day 8 after CsA treatment in the Response group (Figure 4A) but not in the Nonresponse group (Figure 4B). The assay of ECAR was performed and revealed that an enhancement of glycolysis was also observed in neutrophils under in vitro stimulation with CsA from the Response group but not from the Nonresponse group (Figure 4C). To investigate antibacterial ability of neutrophils under stimulation with CsA, we measured expression of antimicrobial peptides as well as ROS and MPO production, and found that the levels of antimicrobial peptides including S100A8 and S100A9 (Figure 4D,E), ROS and MPO (Figure 4F) released from neutrophils were slightly reduced on day 8 after CsA treatment in ASUC patients from the Response group (Figure 4D,F) compared with those in the Nonresponse group (Figure 4E,F). Consistently, the levels of ROS, MPO (Figure 4G), and antimicrobial peptides including S100A8 and S100A9 (Figure 4H) released from neutrophils stimulated with CsA in vitro were decreased significantly compared with controls. Collectively, these data indicate that CsA promotes HIF‐1α expression and glycolysis but suppresses the production of antibacterial peptides, ROS, and MPO by neutrophils in ASUC patients from the Response group but not from the Nonresponse group.

### 3.4 CsA restricts excessive activation of neutrophils in a SIRT6–HIF‐1α‐dependent pathway

**FIGURE 5:**
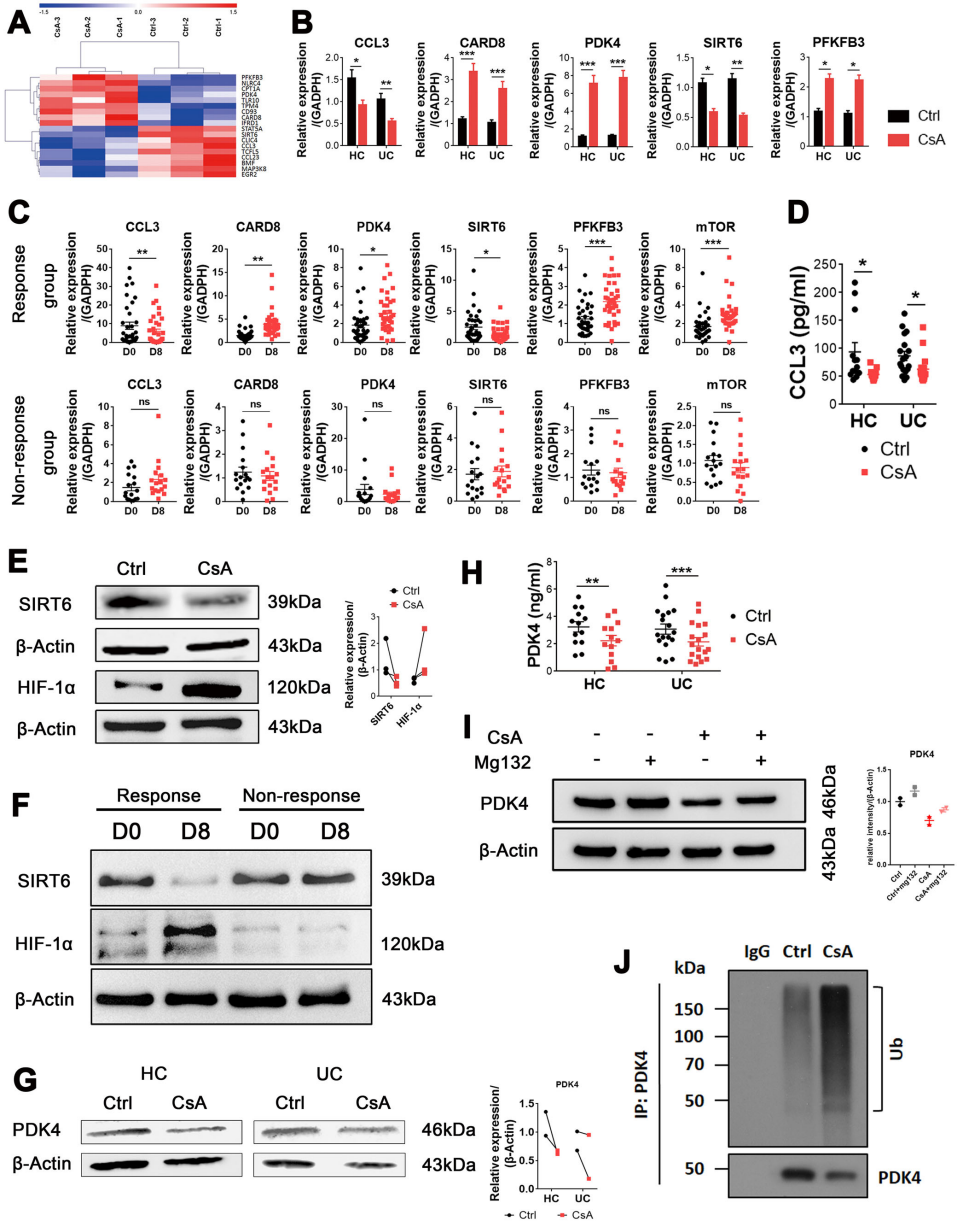
Alteration of transcriptome profiles of neutrophils by cyclosporine A (CsA). (A) Neutrophils (5 × 10^6^ cells) isolated from peripheral blood of acute severe ulcerative colitis (ASUC) patients (*n* = 3) were pretreated in vitro in the absence (Ctrl) or CsA (10 μg/ml) for 3 h and utilized to detect transcriptome differences by RNA sequencing. Heat maps of indicated differential genes in neutrophils pretreated with or without CsA by RNA sequencing. (B) Neutrophils (5 × 10^6^ cells) were isolated from ASUC patients (*n* = 27) and healthy donors (HC, *n* = 25) and cultured, as described in (A). qRT‐PCR was used to quantify the expression of the indicated genes and normalized to GAPDH. (C) Neutrophils (5 × 10^6^ cells) were isolated from ASUC patients of the Response group (*n* = 36) and the Nonresponse group (*n* = 17) before (D0) and on day 8 (D8) after treatment of CsA, and expression of the indicated genes in cultured neutrophils was analyzed by qRT‐PCR. (D) Neutrophils (5 × 10^6^ cells/ml) were isolated from ASUC patients (*n* = 18) and HC (*n* = 13), and cultured, as described in (A), and the supernatants were then harvested after 6 h of culture. The levels of CCL3 were measured by ELISA. (E) Neutrophils (5 × 10^6^ cells/ml) were isolated from ASUC patients (*n* = 3), and cultured for 6 h, as indicated in (A). The levels of SIRT6 and HIF‐1α were measured by WB, and quantification of SIRT6 and HIF‐1α expression is shown in the right panel. (F) Neutrophils (5 × 10^6^ cells/ml) were isolated from ASUC patients of the Response group and the Nonresponse group before (D0) and on day 8 (D8) after treatment of CsA, and the levels of SIRT6 and HIF‐1α were measured by WB. (G and H) Neutrophils (5 × 10^6^ cells/ml) were isolated from ASUC patients and healthy controls, and cultured for 6 h, as indicated in (A). Cells and supernatants were then harvested, and the levels of PDK4 were measured by WB (G) and ELISA (H), respectively. (I) Neutrophils (5 × 10^6^ cells/ml) were isolated from ASUC patients and preincubated with MG‐132 (20 μM) in the presence or absence of CsA (10 μg/ml) for 6 h. Expression of PDK4 was detected by WB. (J) Neutrophils (5 × 10^6^ cells/ml) isolated from ASUC patients were stimulated with or without CsA (10 μg/ml) in vitro for 6 h, and the PDK4 ubiquitination was detected by anti‐Ub immunoblotting. Statistical significance was assessed by two‐tailed Student's *t*‐tests and Tukey's test; *p < .05, **p < .01, ***p < .001, and ****p < .0001; ns, not significant. Data are representative of three independent experiments

Since CsA has been shown to suppress T‐cell activation by inhibiting activation of NF‐AT,^10^ we then wanted to know whether CsA could affect neutrophils in the same pathway. Of note, no differences in the levels of NF‐AT and threonine kinase (Akt) were observed in neutrophils in the presence or absence of CsA when analyzed by qRT‐PCR (Figure S5A) and Western blot (WB) (Figure S5B), respectively, suggesting that the underlying mechanisms whereby CsA regulates neutrophil functions may be different from those in T cells. To address this matter, we isolated neutrophils from peripheral blood of ASUC patients and performed RNA sequencing to investigate the differential gene profiles in neutrophils under stimulation with or without CsA. Gene ontology enrichment and pathway analysis revealed that the differential gene expression was associated with apoptotic process, metabolic process, immune response, cytoskeleton, protein polyubiquitination, and autophagy (Figure S6). We then focused on the genes related to immune regulation and metabolism of neutrophils based on the heatmap analysis, and found that CsA markedly inhibited expression of SIRT6 and chemokine (C–C motif) ligand 3 (CCL3) in neutrophils, but promoted the production of fructose‐2,6‐biphosphatase 3 (PFKFB3), PDK4, and caspase recruitment domain‐containing protein 8 (CARD8) (Figure 5A), which was further verified by qRT‐PCR (Figure 5B). Interestingly, we found that expression of SIRT6 and CCL3 mRNA markedly decreased, while the levels of PFKFB3, PDK4, and CARD8 mRNA increased in neutrophils from the Response group but not from the Nonresponse group (Figure 5C). Importantly, increased expression of CARD8 was associated with the downregulation of apoptosis (Figure 2), which was consistent with previous report.^38^ CCL3, known as macrophage inflammatory protein 1‐α (MIP‐1α), is reported to be involved in the recruitment and activation of neutrophils and macrophages.^39^, ^40^ Thus, Transwell assay revealed that neutrophils significantly promoted the migration of macrophages, whereas this change was obviously retarded in the presence of CsA (Figure S7A). Consistently, immunohistochemical staining of CD68 further confirmed that CsA would significantly attenuate the attraction of neutrophils to macrophages in intestinal mucosa (Figure S7B).

It has been shown that the decrease of SIRT6 and the increase of PFKFB3 and PDK4 are associated with an increase of glycolysis in neutrophils.^41^, ^42^, ^43^ Moreover, HIF‐1α is activated by the mechanistic target of rapamycin (mTOR) but suppressed by SIRT6.^41^, ^42^, ^44^ It not only functions as a classical metabolism regulator to induce the expression of PDK4, leading to downregulating mitochondrial respiration, but also activates the expression of PFKFB3, contributing to glycolysis.^42^, ^45^ Consistent with the mRNA expression of HIF‐1α, the mRNA expression of mTOR in neutrophils was markedly increased in the presence of CsA (Figure 5C). Moreover, the protein levels of CCL3 and SIRT6 were markedly decreased, whereas HIF‐1α was increased in neutrophils when stimulated with CsA (Figure 5D,E). In addition, we found that the protein level of SIRT6 was markedly decreased, while the HIF‐1α was significantly increased in neutrophils from the Response group but not from the Nonresponse group (Figure 5F). However, the protein levels of PDK4 were found to be decreased in neutrophils when stimulated with CsA (Figure 5G,H). We hypothesized this might be related to the posttranscriptional regulation of PDK4.^46^ To clarify this, we then utilized Mg132, an ubiquitination inhibitor, and identified that the posttranscriptional regulation of PDK4 was indeed involved (Figure 5I). The ubiquitination of PDK4 was detected by anti‐Ub immunoblotting and confirmed that the posttranscriptional ubiquitination of PDK4 caused downregulation of PDK4 at the protein level (Figure 5J). Besides, the expression of PDK2 mRNA was not influenced in neutrophils by CsA in vitro (Figure S8A) or from CsA‐treated patients (Figure S8B). And the expression of PDK1 and PDK3 mRNA in neutrophils was relatively low (data not shown). Furthermore, the reduction of PDK4 protein levels led to the decrease of the phosphorylation of pyruvate dehydrogenase (PDH) E1α subunit (Figure S8C). Taken together, these data indicate that CsA‐induced alternations of immune regulatory functions in neutrophils are mediated at least partially by an increase in glycolysis.

### 3.5 CsA promotes the glycolysis and TCA cycle in neutrophils

**FIGURE 6:**
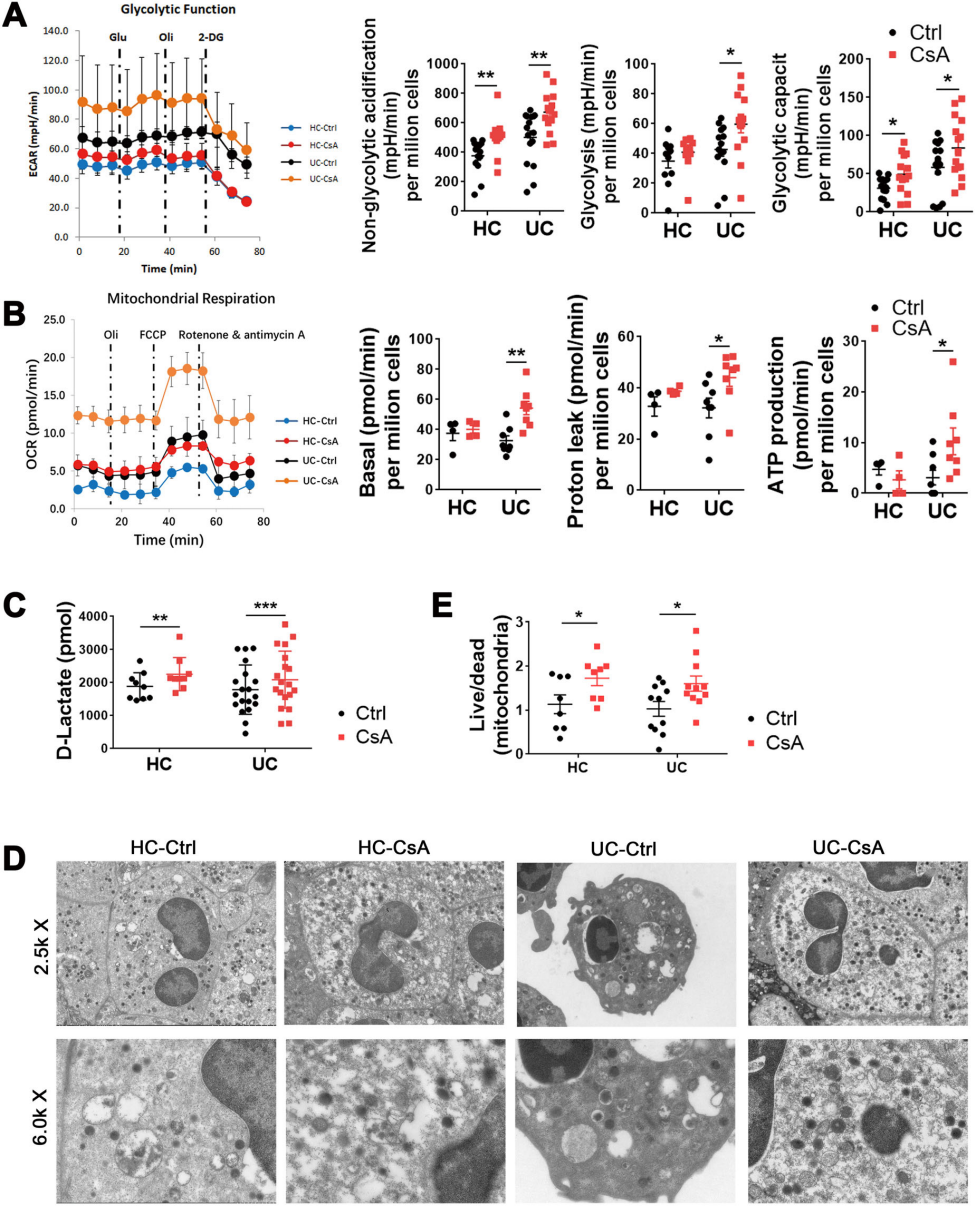
Cyclosporine A (CsA) promotes glycolysis and the TCA cycle of neutrophils. (A–E) Neutrophils (1 × 10^6^ cells/ml) were isolated from UC patients and healthy controls (HC), cultured as indicated in Figure 5A, and prepared for further experiments. (A and B) The dynamic changes of glycolysis and TCA cycle were measured by ECAR (A; HC, *n* = 14; UC, *n* = 16) and OCR (B; HC, *n* = 4; UC, *n* = 8), respectively, using a Seahorse extracellular flux analyzer. Quantitative analysis of ECAR and OCR is shown in the right panels. (C) Quantitative analysis of lactate (HC, *n* = 9; UC, *n* = 19) was performed by Fluorometric Assay Kit. (D) Representative scanning electron micrographs of neutrophils (HC, *n* = 8; UC, *n* = 11) and mitochondria under magnification ×2.5k (upper panels) and ×6.0k (lower panels), respectively. (E) Quantitative analysis of the ratio of survival mitochondria to apoptotic mitochondria in each field of vision according to the data from (D). Statistical significance was assessed by two‐tailed Student's *t*‐tests; *p < .05, **p < .01, ***p < .001, and ****p < .0001; ns, not significant

To evaluate the consumption mode of glucose in neutrophils, we performed the assay of ECAR and oxygen consumption rate (OCR) to determine the dynamic changes of glycolysis and TCA cycle, respectively. The results demonstrated that CsA promoted the glycolysis (Figure 6A) and the TCA cycle (Figure 6B). Quantitative analysis of ECAR and OCR, including glycolytic capacity, proton leak, and adenosine triphosphate (ATP) production, further confirmed these findings (Figure 6A,B). In addition, the quantitative assay of the final product lactate further proved that CsA increased the production of D‐lactate and glycolysis in neutrophils, but did not influence the production of L‐lactate in neutrophils (Figure 6C, Figure S9). We took advantage of transmission electron microscopy (TEM) to determine the morphological changes of neutrophils and mitochondria, and observed that CsA decreased the apoptosis of neutrophils, as manifested by the suppression of the progress of nuclear pyknosis, nuclear fragmentation and nuclear dissolution, and preserved normal mitochondria and less architecture changes, as characterized by more and longer cristae and less swelling, compared with untreated neutrophils (Figure 6D,E).

### 3.6 Blockade of SIRT6 and HIF‐1α regulates the apoptosis and migration of CsA‐treated neutrophils

**FIGURE 7:**
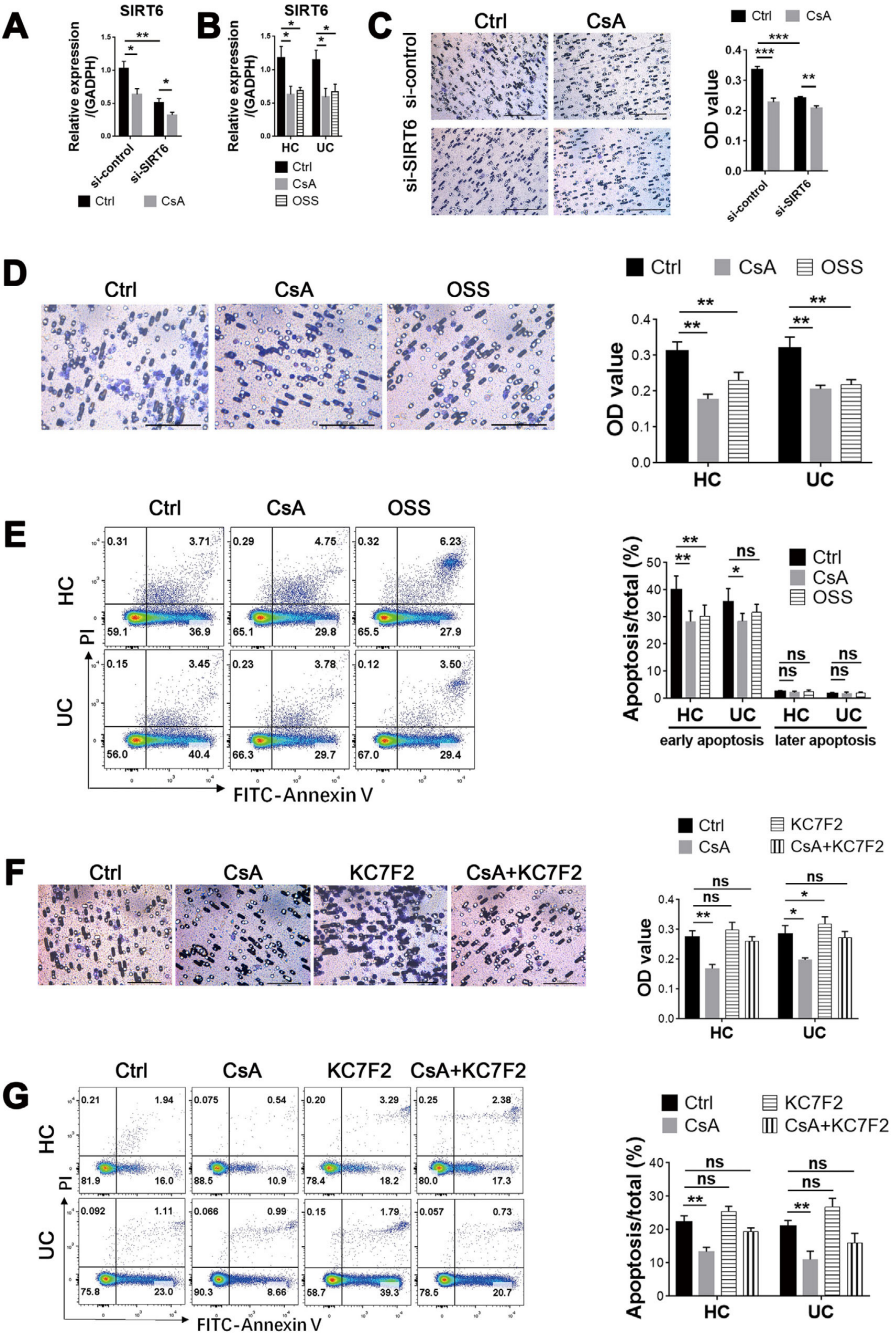
Blockage of SIRT6 and HIF‐1α inhibits the migration of neutrophils and regulates the activation of neutrophils. (A) HL‐60 cells (1 × 10^6^ cells) were transfected with SIRT6‐specific siRNA or the scramble siRNA and then treated with cyclosporine A (CsA) (10 μg/ml) for 3 h, and the level of SIRT6 mRNA was determined by qRT‐PCR. (B) Neutrophils (5 × 10^6^ cells) isolated from peripheral blood of acute severe ulcerative colitis (ASUC) patients (*n* = 8) and healthy controls (HC, *n* = 10) were pretreated with OSS_128167 (an inhibitor of SIRT6, 100 μM/ml) in vitro for 3 h, and the level of SIRT6 mRNA was determined by qRT‐PCR. (C and D) HL‐60 cells (C; 1 × 10^6^ cells) or neutrophils (D; 5 × 10^6^ cells) isolated from healthy donors (*n* = 10) and naïve patients with active UC (*n* = 8) were cultured, as indicated in (A,B). The migration of HL‐60 cells and neutrophils was detected, as described in Figure 3A, and the quantification is shown in the right panel. Scale bars: 100 μm. (E) Neutrophils (1 × 10^6^ cells) were isolated and cultured, as indicated in (D), and the representative images of apoptotic neutrophils stained with annexin V were measured by flow cytometry. The percentages of both early (annexin V^+^/PI^−^) and later (annexin V^+^/PI^+^) apoptotic neutrophils were counted and are shown in the right panel. (F and G) Neutrophils (5 × 10^6^ cells) isolated from peripheral blood of ASUC patients (*n* = 8) and healthy controls (HC, *n* = 10) were pretreated with KC7F2 (an inhibitor of HIF‐1α, 20 μM/ml) in vitro for 3 h. (F) The migration of HL‐60 cells and neutrophils was detected as described in Figure 3A, and the quantification is shown in the right panel. Scale bars: 100 μm. (G) The representative images of apoptotic neutrophils stained with annexin V were measured by flow cytometry. The percentages of apoptotic neutrophils were statistically analyzed and are shown in the right panel. Statistical significance was assessed by Tukey's test; *p < .05, **p < .01, ***p < .001, and ****p < .0001; ns, not significant

Given that the SIRT6–HIF‐1α signaling pathway plays a critical role in regulating glucose metabolism,^37^, ^41^ we hypothesized that the metabolism and functional changes of neutrophils stimulated by CsA could be mediated by the SIRT6–HIF‐1α pathway instead of the Akt–mTOR pathway, implying that CsA may suppress SIRT6 but enhance HIF‐1α in neutrophils. To this end, HL‐60 cells, a human promyelocytic leukemia cell line, were cultured and transfected with *SIRT6*‐specific siRNA to block the expression of SIRT6, and then treated with or without CsA for 3 h. Neutrophils isolated from ASUC patients and HCs were cultured and stimulated in vitro with OSS‐128167 (an inhibitor of SIRT6), KC7F2 (an inhibitor of HIF‐1α), and DCA (an inhibitor of PDK4), respectively, for 3 h. We found that blockade of SIRT6 could regulate the expression of HIF‐1α and PDK4, and thus significantly inhibited the migration of neutrophils, but did not influence both the early and later apoptosis of neutrophils compared with controls (Figure 7A–E, Figure S10A–C), suggesting that blockage of SIRT6 had, to some extent, the same effects on the functions of neutrophils as CsA did. In contrast, inhibition of HIF‐1α by KC7F2 markedly increased the migration of neutrophils as well as expression of PDK4, which was opposite to the effect of CsA (Figure 7F, Figure S10A–C). Additionally, blockade of HIF‐1α did not affect the apoptosis of neutrophils (Figure 7G). However, the inhibition of PDK4 with DCA did not alter the functional alternations of neutrophils (Figure S11A,B), which could be ascribed to the facts that the glucose metabolism of neutrophils is dominated by glycolysis and that the effect of TCA cycle on its function is compromised. Besides, inhibition of glycolysis by 2‐deoxy‐D‐glucose (2‐DG) markedly increased the migration and this process could be suppressed by CsA, suggesting that increased glycolysis is essential for the CsA‐induced functional alternations of neutrophils (Figure S12A,B). Collectively, these data indicate that the SIRT6–glycolysis signaling pathway plays a critical role in modulating functional changes of CsA‐treated neutrophils.

## 4 DISCUSSION

In this study, we investigated the underlying mechanisms of CsA in the treatment of ASUC and the regulation of neutrophil homeostasis, and found that CsA could restrict excessive neutrophil activation. Specifically, CsA unequivocally increased neutrophil HIF‐1α expression and glycolysis, while it significantly suppressed the abilities of migration, apoptosis, as well as the release of ROS, MPO, antibacterial peptide, and IL‐8 of neutrophils. RNA sequencing revealed that CsA modulated neutrophil properties including apoptotic process, metabolic process, immune response, cytoskeleton, protein polyubiquitination, and autophagy. Altered gene expression in neutrophils by CsA, including glycolysis‐associated SIRT6, the upstream signaling molecules of HIF‐1α, as well as its downstream signaling molecules PFKFB3 and PDK4, could fuel neutrophil glycolysis and TCA cycle, which thus align with inhibiting the excessive activation of neutrophils. Taken together, our data indicate that restriction of excessive neutrophil activation via the SIRT6–HIF‐1α‐dependent glycolysis pathway contributes to clinical remission in severe UC.

Neutrophils are considered to function as double‐edged swords in exerting restorative actions as well as fueling inflammation.^14^ In the early stage of inflammation, neutrophils are activated and cross intestinal epithelial barriers to restrain mucosal inflammation.^17^, ^23^ During the advanced phases of inflammatory response, however, excessive neutrophil activation, infiltration, and subsequent apoptosis lead to the disruption of intestinal barrier integrity and sustaining release of proinflammatory signals, cause collateral tissue damage, and delay the recovery.^14^, ^17^ In our current study, we found that CsA significantly decreased IL‐8, ROS and MPO production by neutrophils and suppressed the capacity of migration as well as apoptosis of neutrophils in acute stage of inflammation concomitant with the alleviation of intestinal mucosal inflammation. According to RNA sequencing, the mRNA levels of CCL3, which involves in the recruitment and activation of neutrophil and macrophages,^39^, ^40^ were found to be decreased while the mRNA levels of CARD8, which involves in the negative regulation of apoptosis and inflammation,^38^ were increased in CsA‐primed neutrophils compared with untreated neutrophils. Collectively, our data thus indicate that CsA restricts the migration and apoptosis of neutrophils. Therefore, this regulation not only increases the retention of neutrophils at inflammatory site for sterilization, but also strengthens circulatory lifespan of neutrophils and cumulative dwell time in the intestine at a quite steady state, thus restricting tissue damage caused by excessive activation and infiltration of external neutrophils and eventually maintaining intestine homeostasis.

Neutrophils have powerful effects on antimicrobial abilities through the phagocytosis, degranulation, and the release of ROS and antimicrobial peptides, which involve in the regulation of autoimmunity and pathological processes including dermatologic disease, infection, chronic inflammation, systemic lupus erythematosus, and vasculitis.^20^, ^27^, ^47^ Our studies unequivocally demonstrated that the release of ROS, MPO, and antimicrobial peptides (including S100A8, S100A9) was markedly inhibited in CsA‐primed neutrophils, which may be contributed to the compensatory regulation of neutrophil functions, as ROS production is implicated to be associated with tissue damage.^47^ Taken together, tissue damage induced by excessively activated neutrophils is modified via the decrease of migration, apoptosis, and ROS production, which prevents excessive activation and inflammatory outbreaks of recruited neutrophils in gut mucosa.

In the past decades, CsA has been thought to reduce the dephosphorylation of NF‐AT via suppressing the function of calcineurin and thus inhibit NF‐AT anchored to its target genes.^10^ We took advantage of RNA sequencing to assess the differential gene profiles of neutrophils in response to CsA and illustrate that CsA could induce significant changes in apoptotic process, metabolic process, regulation of immune response, cytoskeleton, protein polyubiquitination, and autophagy. Particularly, the mRNA levels of CCL3 and SIRT6 significantly decreased, while CARD8, PDK4, and PFKFB3 mRNA expressions markedly increased. Although previous study reported that SIRT6 regulates the function of pancreatic cancer cells by modulating the NF‐AT activity.^48^ However, we found that CsA could balance neutrophil immune functions via SIRT6 by increasing expression of HIF‐1α but not the activity of NF‐AT. SIRT6 could inhibit the activation of HIF‐1α,^41^ regulate its downstream molecules including PDK4 and PFKFB3, and modulate glycolysis and TCA cycle of neutrophils.^42^, ^45^ Previous data have shown that glucose consumption mode, which is highly regulated by HIF‐1α,^4^ is closely related to immune homeostasis and intestinal mucosal epithelial damage repair.^49^, ^50^ Moreover, HIF‐1α activation is reported to reduce neutrophil apoptosis and fastens activated neutrophils at inflammatory site for sterilization.^26^, ^51^ Thus, the decrease of SIRT6 expression and the enhancement of downstream genes including HIF‐1α, PDK4, and PFKFB3 in neutrophils by CsA subsequently contribute to the increase of glycolysis and TCA cycle, and modulate the immune functions of neutrophils, thereby fine‐tuning neutrophil homeostasis. The assay of ECAR and OCR, the measurement of key metabolites, and TEM further confirmed that CsA could facilitate glycolysis and TCA cycle of neutrophils via the SIRT6–HIF‐1α–glycolysis pathway, thus maintaining neutrophil homeostasis in intestinal mucosa. Nevertheless, we still envisaged to perform experimental validation in *Sirt6*‐deficient mice. However, *Sirt6*‐deficient mice have a really short lifespan, which prevents us from validating the hypothesis in vivo.^41^, ^52^ Thus, we utilized an inhibitor to restrain important nodes at the SIRT6 pathway. Importantly, we found that blockade of SIRT6 in neutrophils or HL‐60 cells had the same effects as CsA did, while inhibition of HIF‐1α demonstrated the opposite effects and its effect on PDK4 inhibition was weaker than CsA. In addition, inhibition of glycolysis by 2‐DG markedly increased the migration, and this process was suppressed by CsA. These results provide compelling evidence to show that CsA suppresses excessive mucosal inflammation in ASUC patients by inhibiting SIRT6 expression, subsequently modulating its downstream pathways, and fueling neutrophil glycolysis and TCA cycle to restrict excessive activation of neutrophils and maintain intestinal homeostasis.

## 5 CONCLUSION

**FIGURE 8:**
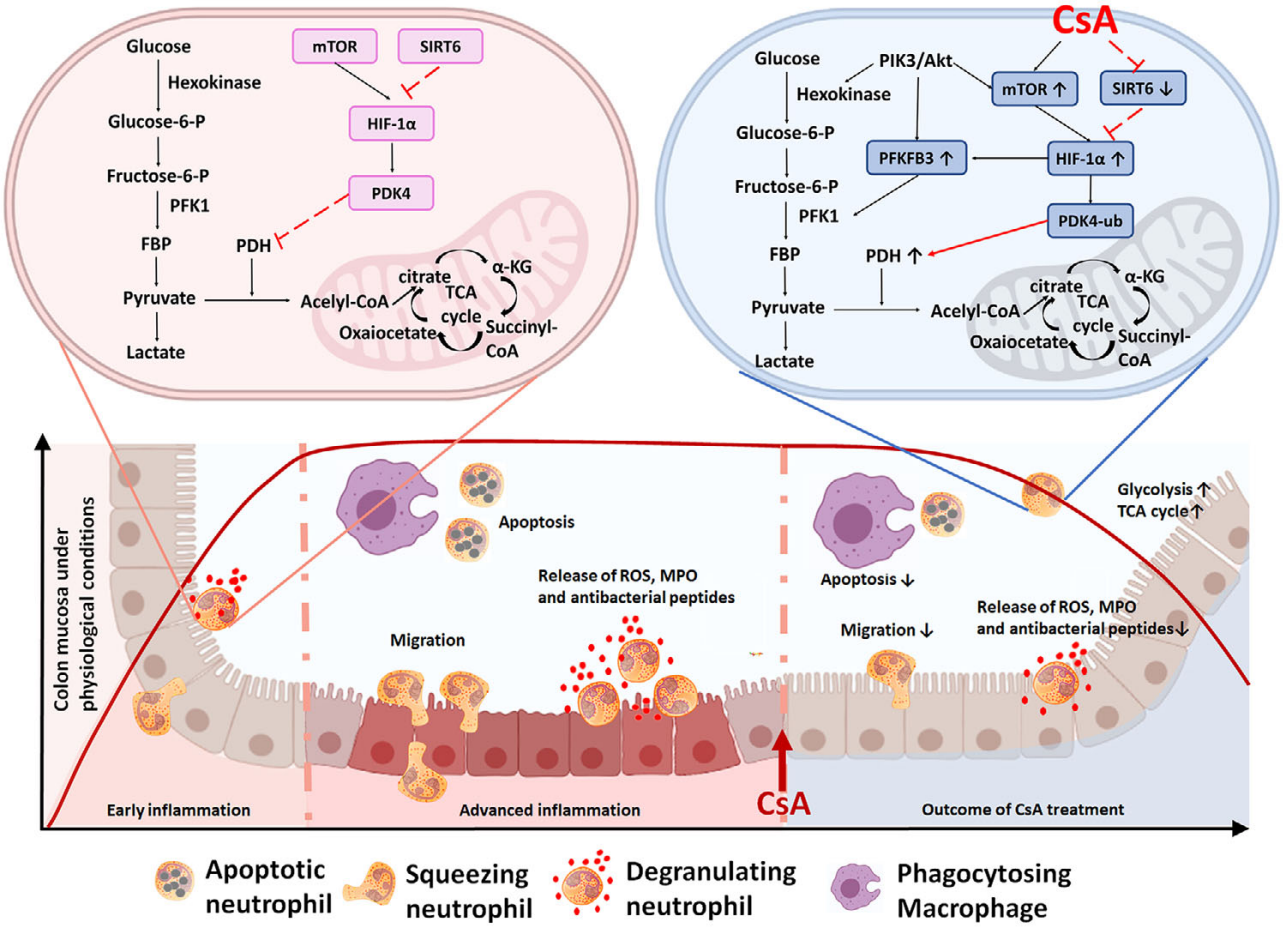
Cyclosporine A (CsA) restricts excessive activation of neutrophils and enhances glycolysis by promoting HIF‐1α and SIRT6 expression. This schematic shows current understanding of how CsA restricts excessive activation of neutrophils and maintains mucosal homeostasis by fueling the glycolysis and TCA cycle of neutrophils during acute severe ulcerative colitis (ASUC). Under physiological conditions, HIF‐1α activates PDK4, which then blocks the TCA cycle by inhibiting the activation of pyruvate dehydrogenase (PDH). When administrated during ASUC, CsA promotes glycolysis by enhancing expression of PFKFB3 via the activation of HIF‐1α but not Akt. Moreover, CsA also facilitates mTOR expression but suppresses SIRT6 expression, which further promotes HIF‐1α expression and preferentially ubiquitinates and degrades PDK4, leading to an increase of TCA cycle

In conclusion, restriction of neutrophil excessive activation by CsA plays a critical role in maintaining intestinal homeostasis and controlling overloaded proinflammatory response. Mechanistically, CsA suppresses SIRT6 expression and subsequently fuels HIF‐1α expression as well as glycolysis and TCA cycle in neutrophils, thereby modulating the properties in the context of anti‐inflammatory stigma. Therefore, the effects of CsA on neutrophil via the SIRT6–HIF‐1α‐dependent glycolysis pathway may be an alternative pathway of its anti‐inflammatory effects, which is different from its effect on T cells (Figure 8). Our study provides a great potential for novel therapeutic approach in UC patients through modulating neutrophil functions by inhibiting SIRT6 expression. Importantly, these emerging findings unveil an important pathway through restricting excessive activation of neutrophils by CsA in regulating mucosal innate immunity and highlight a novel therapeutic strategy in the management of UC.

## AUTHOR CONTRIBUTIONS

Zhanju Liu conceived and designed the study. Huiying Lu and Jian Lin performed all the experiments and analyzed the data. Huiying Lu, Chunjin Xu, Keqiang Zuo, Xiaoping Zhang, Mingsong Li, Hailiang Huang, Zhong Li, Baisui Feng, and Zhanju Liu interpreted the data. Wei Wu, Mingming Sun, Chunjin Xu, and Baisui Feng enrolled patients, maintained clinical dataset, and collected specimens. Huiying Lu and Zhanju Liu drafted the manuscript. All the authors have approved the final version of this article.

## ETHICS APPROVAL AND CONSENT TO PARTICIPATE

Ethical approval was obtained from Ethics Committee of the Shanghai Tenth People's Hospital of Tongji University (SHSY‐IEC‐4.0/19‐52/01).

## CONFLICT OF INTEREST

The authors declare that there is no conflict of interest.

## Supporting information

Supporting Information

## Data Availability

The data used to support the findings of this study are openly available in the article and the supplementary information file.
