# Supplementary material for: Cyclosporine modulates neutrophil functions via the SIRT6–HIF‐1α–glycolysis axis to alleviate severe ulcerative colitis
Source: Clin Transl Med. 2021 Feb 14;11(2):e334. doi: 10.1002/ctm2.334 (PMC7882115; doi:10.1002/ctm2.334)
Supplement: Supplementary file 1 — Supporting Information [file CTM2-11-e334-s001.docx]

**Supplementary Tables**

**Supplementary Table 1 The characteristics of UC patients and healthy donors in *in vitro* experiment**

|  | HC (n = 25) | UC (n = 27) |
| --- | --- | --- |
| Age (years)  Gender  Male  Female  Duration of disease (months) | 18-45  13  12 | 21-62  15  12  2-128 |
| Therapy |  |  |
| Mesalazine  Corticosteroids  Lesion location^*^ |  | 19  17 |
| E1  E2  E3 |  | 3  6  18 |
| Lichtiger score |  | 11.26 ± 1.34 |
| CRP (mg/L) |  | 32.17 ± 43.34 |
| Hemoglobin (g/L) |  | 115.22 ± 13.63 |
| WBC (×10^9^) |  | 8.98 ± 2.98 |
| RBC (×10^12^) |  | 4.07 ± 0.48 |
| PMN (×10^9^) |  | 6.37 ± 2.89 |
| PMN (%) |  | 69.19 ± 9.98 |

^*^According to the Montreal classification.

**Supplementary Table 2 Primers used to amplify the coding and adjacent non-coding sequences of genes**

| **Gene name** | **Forward primer** | **Reverse primer** |
| --- | --- | --- |
| GAPDH (Hu) | GGAGCGAGATCCCTCCAAAAT | GGCTGTTGTCATACTTCTCATGG |
| NF-AT (Hu) | GCTGGATAACAGTCGGATGTC | GCCTCTGCTTTGGATTTCGTT |
| AKt1 (Hu) | AGCGACGTGGCTATTGTGAAG | GCCATCATTCTTGAGGAGGAAGT |
| AKt2 (Hu) | ACCACAGTCATCGAGAGGACC | GGAGCCACACTTGTAGTCCA |
| AKt3 (Hu) | TGTGGATTTACCTTATCCCCTCA | GTTTGGCTTTGGTCGTTCTGT |
| MPO (Hu) | TGCTGCCCTTTGACAACCTG | TGCTCCCGAAGTAAGAGGGT |
| mTOR (Hu) | ATGCTTGGAACCGGACCTG | TCTTGACTCATCTCTCGGAGTT |
| IL-8 (Hu) | TTTTGCCAAGGAGTGCTAAAGA | AACCCTCTGCACCCAGTTTTC |
| S100A8 (Hu) | ATGCCGTCTACAGGGATGAC | ACTGAGGACACTCGGTCTCTA |
| S100A9 (Hu) | GGTCATAGAACACATCATGGAGG | GGCCTGGCTTATGGTGGTG |
| CCL3 (Hu) | AGTTCTCTGCATCACTTGCTG | CGGCTTCGCTTGGTTAGGAA |
| HIF-1α (Hu) | GAACGTCGAAAAGAAAAGTCTCG | CCTTATCAAGATGCGAACTCACA |
| SIRT6 (Hu) | CCCACGGAGTCTGGACCAT | CTCTGCCAGTTTGTCCCTG |
| CARD8 (Hu) | GAAGCGAAACTGCATATTCTGGT | GGGTTGGAAGAGGCATGGC |
| PDK4 (Hu) | GGAGCATTTCTCGCGCTACA | ACAGGCAATTCTTGTCGCAAA |
| PFKFB3 (Hu) | TTGGCGTCCCCACAAAAGT | AGTTGTAGGAGCTGTACTGCTT |
| TNF-α (Hu) | CCTCTCTCTAATCAGCCCTCTG | GAGGACCTGGGAGTAGATGAG |
| TGF-β (Hu) | GGCCAGATCCTGTCCAAGC | GTGGGTTTCCACCATTAGCAC |
| IL-1β (Hu) | ATGATGGCTTATTACAGTGGCAA | GTCGGAGATTCGTAGCTGGA |
| IL-6 (Hu) | ACTCACCTCTTCAGAACGAATTG | CCATCTTTGGAAGGTTCAGGTTG |
| IL-10 (Hu) | GACTTTAAGGGTTACCTGGGTTG | TCACATGCGCCTTGATGTCTG |
| IL-17A (Hu) | TCCCACGAAATCCAGGATGC | GGATGTTCAGGTTGACCATCAC |
| IL-22 (Hu) | GCTTGACAAGTCCAACTTCCA | GCTCACTCATACTGACTCCGT |
| PDK1 | CTGTGATACGGATCAGAAACCG | TCCACCAAACAATAAAGAGTGCT |
| PDK2 | ATGAAAGAGATCAACCTGCTTCC | GGCTCTGGACATACCAGCTC |
| PDK3 | CGCTCTCCATCAAACAATTCCT | CCACTGAAGGGCGGTTAAGTA |

**Supplementary Table 3** **Reagents used in *in vitro* experiments**

| **Reagent** | **Sources** | **Identifier** |
| --- | --- | --- |
| **Antibodies** |  |  |
| Anti-phospho Akt | Cell Signaling Technology | 4060 |
| Anti-total Akt | Cell Signaling Technology | 2920 |
| Anti-HIF-1α | Cell Signaling Technology | 36169 |
| Anti-SIRT6 | Abcam | ab88494 |
| Anti-PDK4 | Abcam | ab89295 |
| Anti-MPO | Abcam | ab208670 |
| Anti-CD4 | Abcam | ab133616 |
| Anti-CD68 | Abcam | ab31630 |
| PDH E1α | Santa Cruz Biotechnology | sc-377092 |
| pS232 | Calbiochem | AP1063 |
| pS293 | Abcam | ab92696 |
| pS300 | Calbiochem | AP1064 |
| **ELISA** |  |  |
| Human Myeloperoxidase Myeloperoxidase | Biolegend | 440008 |
| Human PDK4 | Abcam | ab126582 |
| Human IL-1β | Biolegend | 437006 |
| Human IL-8 | Biolegend | 431506 |
| Human TNF-α | Biolegend | 430206 |
| Human IFN-γ | Biolegend | 437004 |
| Human CCL3 | R&D | PDMA00 |
| Human S100A8/9 | R&D | DS8900 |
| **Other Reagents** |  |  |
| D-Lactate Assay Kit | Biovision | K668-100 |
| L-Lactate Assay Kit | Biovision | K627-100 |

**Supplementary Figure Legends**

**
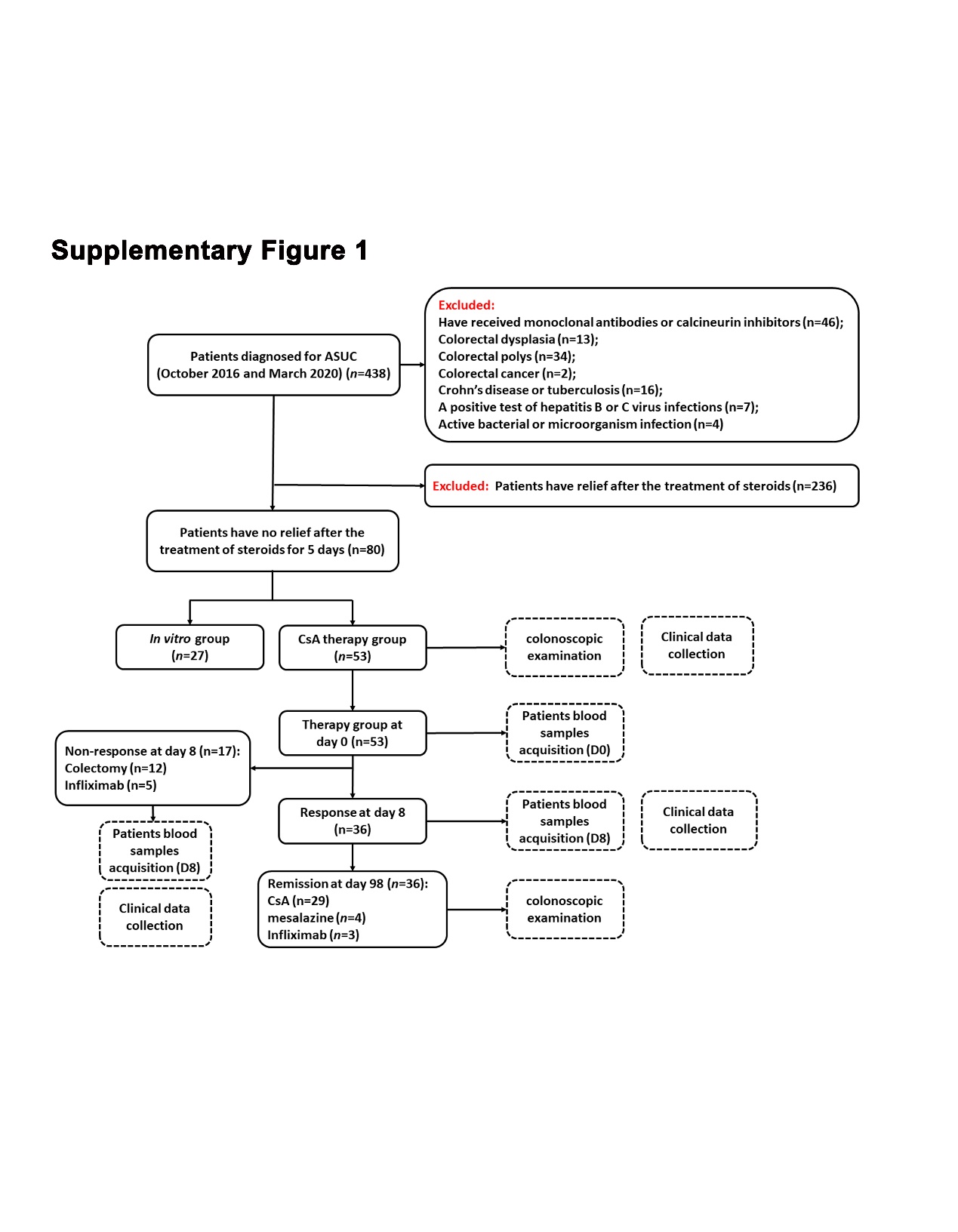
 Fig. S1** Flow diagram of patient selection. Flow diagram for screening ASUC patients with CsA administration and collecting blood samples and clinical data. ASUC, acute severe ulcerative colitis; CsA, cyclosporine A.

**
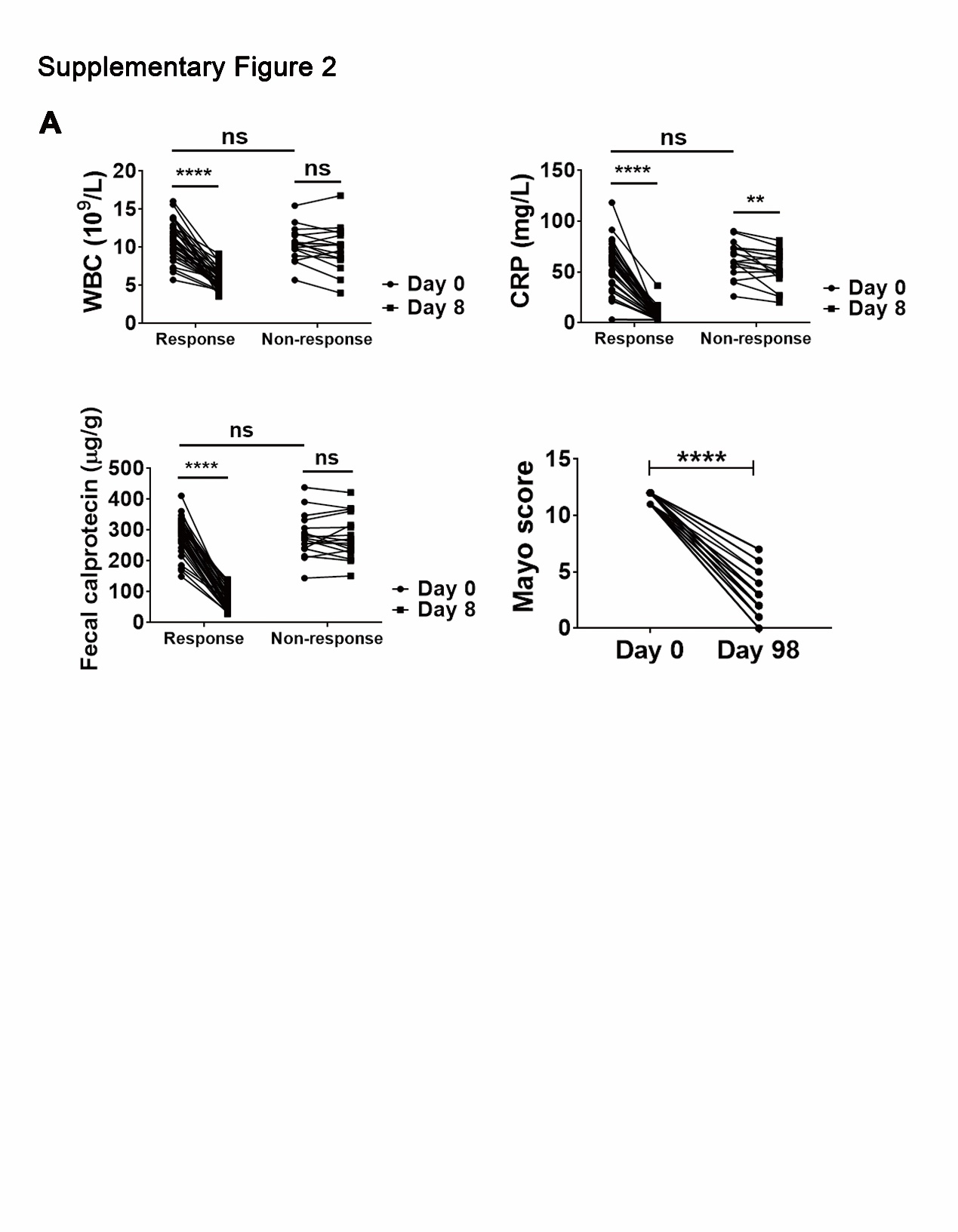
Fig. S2** Changes of clinical parameters of patients with ASUC who underwent CsA treatment. (A) Patients with ASUC received treatment with CsA at the dose of 3 mg/kg/day for 7 days (Response group, n = 36; Non-response group, n = 17), and clinical parameters of these patients including Mayo score, WBC count, CRP, fecal calprotectin were shown. Statistical significance was assessed by two-tailed Student’s *t* tests. *****P* < 0.0001.

**
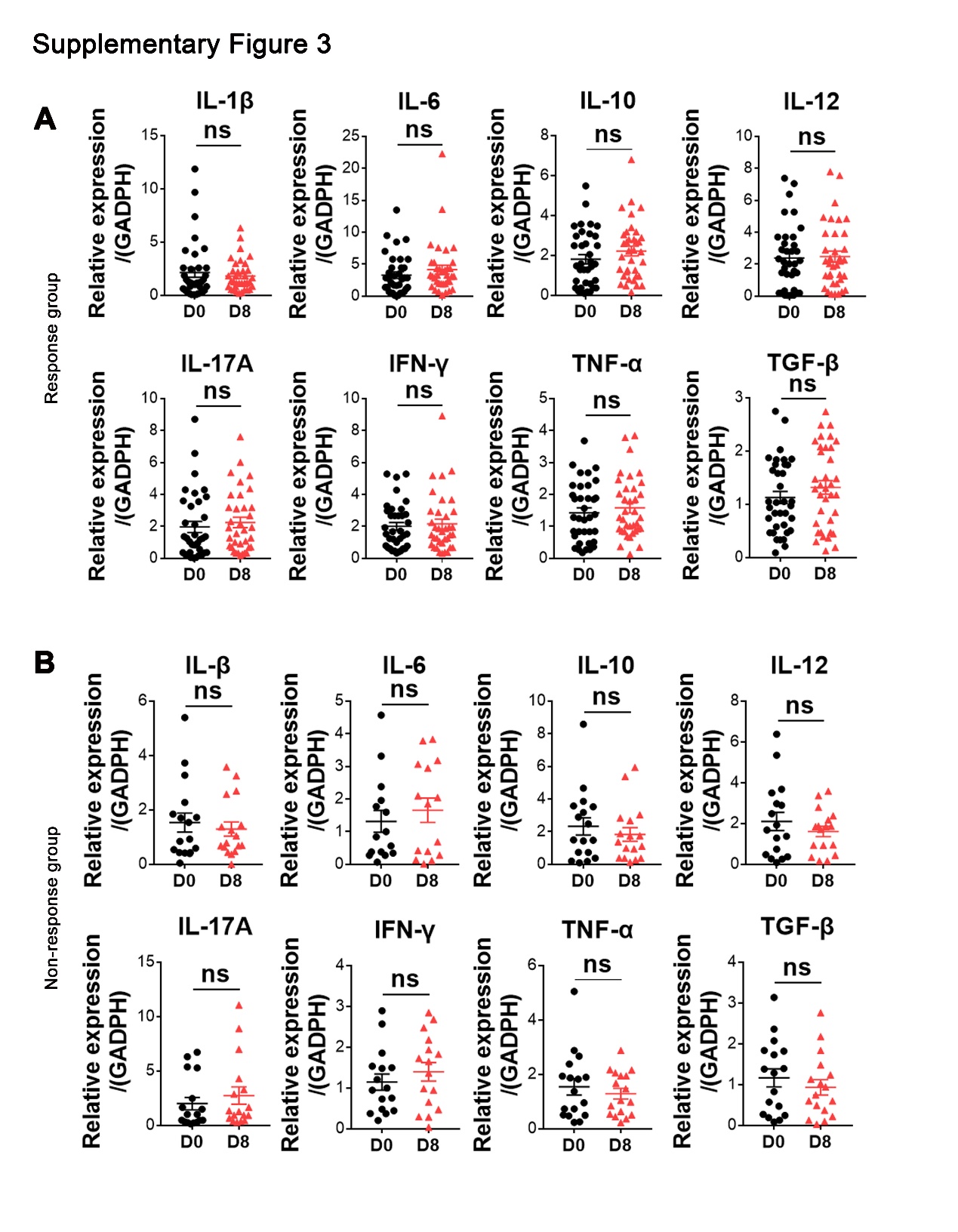
 Fig. S3** mRNA expression of cytokines of neutrophils from patients with ASUC who underwent CsA treatment. (A-B) Neutrophils (5 × 10^6^ cells) were collected and cultured as described in Fig. 4A-B. mRNA expression of cytokines of neutrophils from the Response group (A, n = 36) and Non-response group (B, n = 17) was detected by qRT-PCR. Statistical significance was assessed by Tukey’s test. Mean ± SEM are shown.

**
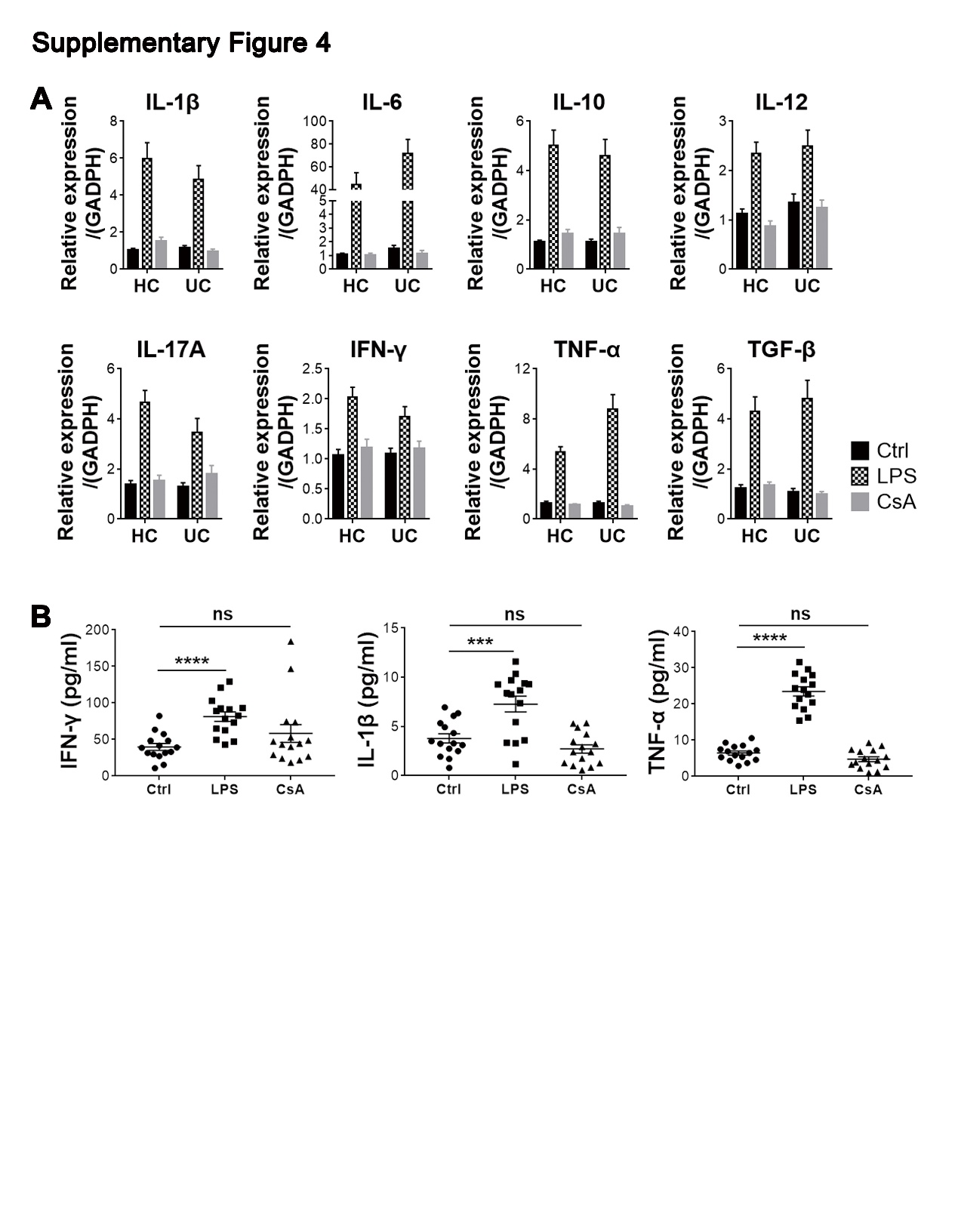
 Fig. S4** Cytokine profiles from neutrophils of ASUC patients under stimulation with CsA *in vitro*. (A-B) Neutrophils (5 × 10^6^ cells/mL) isolated from ASUC patients and healthy donors were pretreated *in vitro* in the absence (Ctrl) or presence of LPS (300 ng/mL) or CsA (10 μg/mL) for 3 hours as indicated in Fig. 3F. Cells and supernatants were then harvested and the levels of cytokines (eg, IL-1β, IL-6, IL-10, IL-12, IL-17A, TNF-α, TGF-β, and IFN-γ) were analyzed by qRT-PCR (A, HC = 25, UC = 27) and ELISA (B, n = 15), respectively. Statistical significance was assessed by Tukey’s test. Mean ± SEM are shown. **P* < 0.05, ***P* < 0.01, ****P* < 0.001, and *****P* < 0.0001; ns, not significant.

**
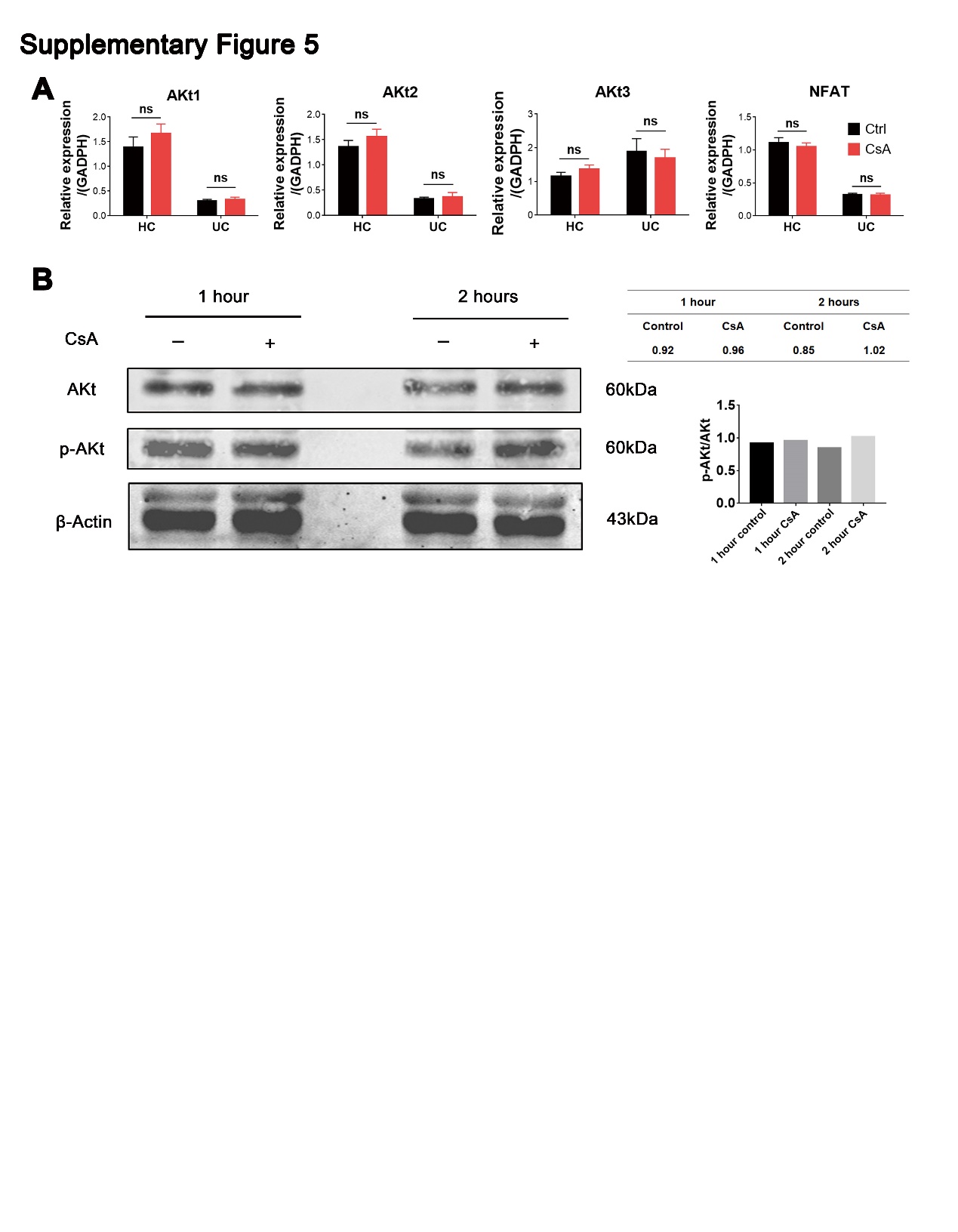
Fig. S5** CsA does not affect the expression of Akt and NF-AT in neutrophils. (A) Neutrophils (5 × 10^6^ cells/mL) isolated from ASUC patients (n = 27) and healthy donors (n = 25) were pretreated *in vitro* in the absence (Ctrl) or presence of CsA (10 μg/mL) for 3 hours as described in Fig. 5B, and expression of NF-AT and Akt was analyzed by qRT-PCR. Statistical significance was assessed by two-tailed Student’s *t* tests. (B) Neutrophils (5 × 10^6^ cells/mL) isolated from ASUC patients were pretreated *in vitro* in the absence (Ctrl) or presence of CsA (10 μg/mL) for 1 or 2 hours. The dephosphorylation of Akt was detected by WB. Mean ± SEM are shown. ns, not significant.

**
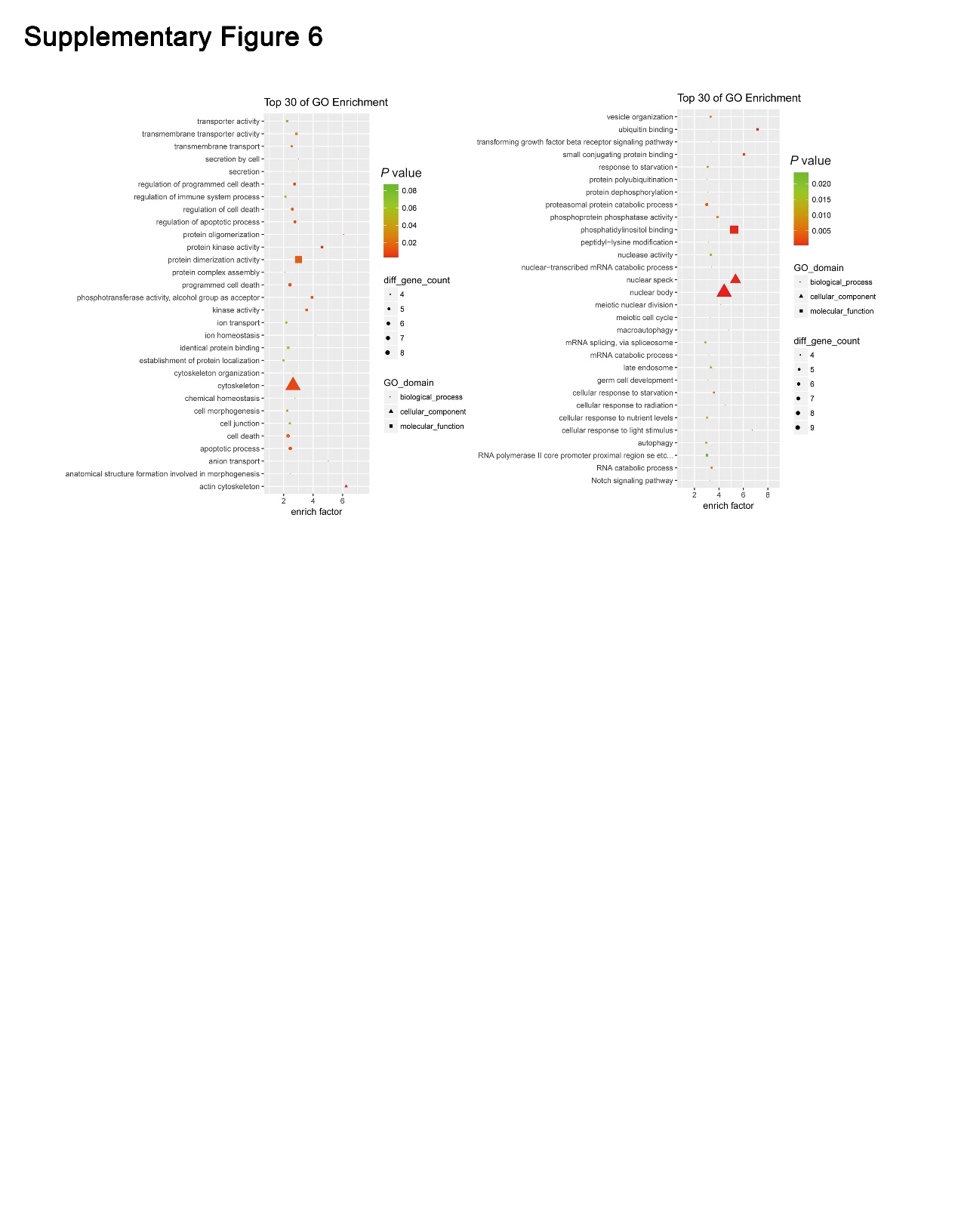
 Fig. S6** Differential functional profiles of neutrophils pretreated with CsA. Neutrophils were isolated, cultured and analyzed as indicated in Fig. 5A (n = 3). Gene Ontology enrichment revealed the differentiation of gene expression profiles.

**
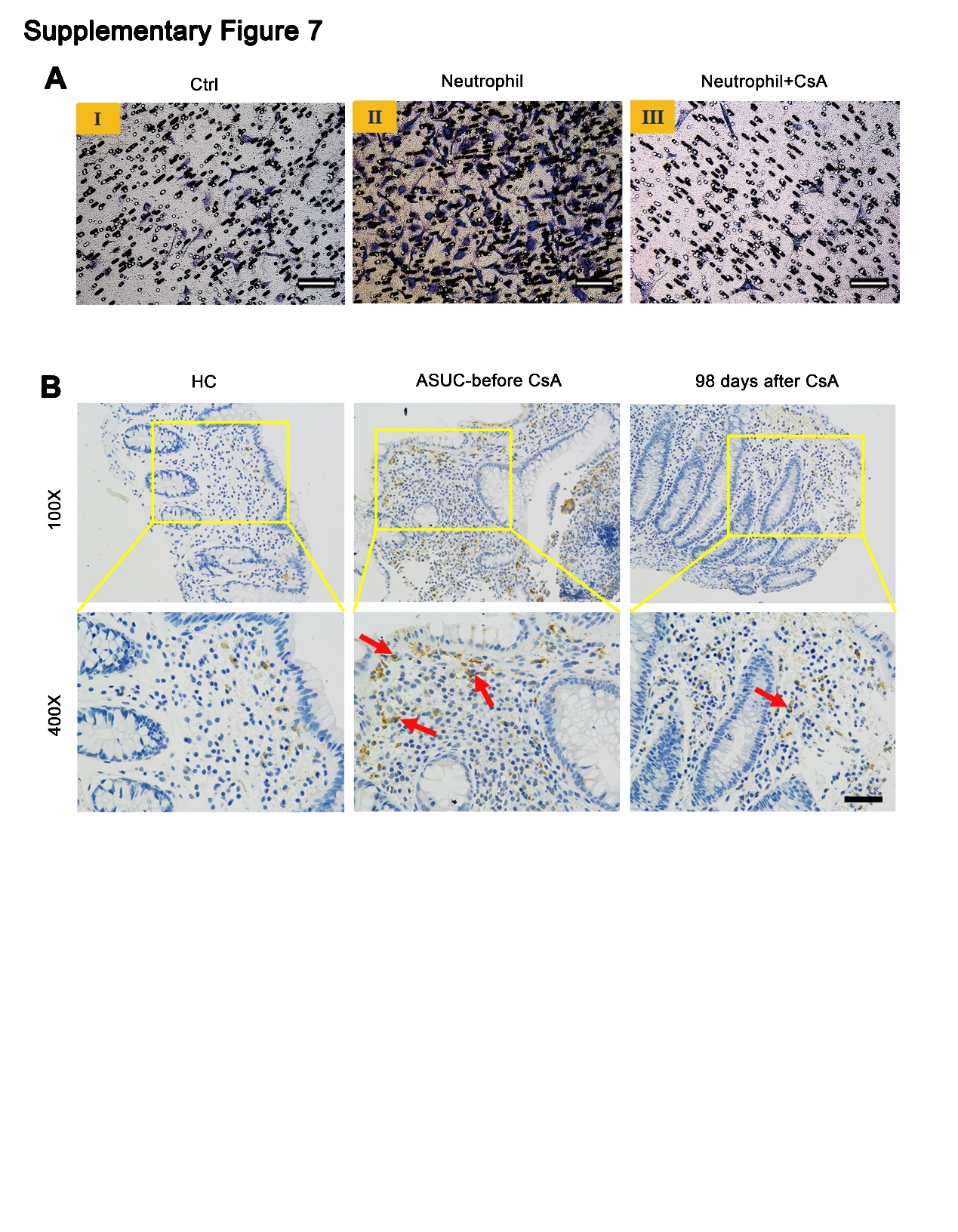
Fig. S7** CsA-primed neutrophils inhibit the migration of macrophage. (A) The migration of macrophages *in vitro* was analyzed using Transwell chamber. Macrophages (1 × 10^5^ cells) extracted and induced from mouse bone marrow were seeded in the upper chamber, and neutrophils isolated from mouse bone marrow were seeded in the lower chamber in the absence (Neutrophil, II) or presence of CsA (10 μg/mL, Neutrophil + CsA, III). Lower chamber without cells or stimulants was set as control group (Ctrl, I). Representative images of staining of macrophages after migration were shown. Scale bars: 100 μm. (B) Intestinal biopsies were collected as described in Fig. 1C, representative images of immunohistochemical staining for CD68 expression, which indicates macrophage infiltration in inﬂamed colon from ASUC patients in the Response group (n = 36, the middle and right panels) and normal colon mucosa from HC (n = 5, the left panels). The arrows indicate macrophages. Scar bar represents 50 μm (lower).

**
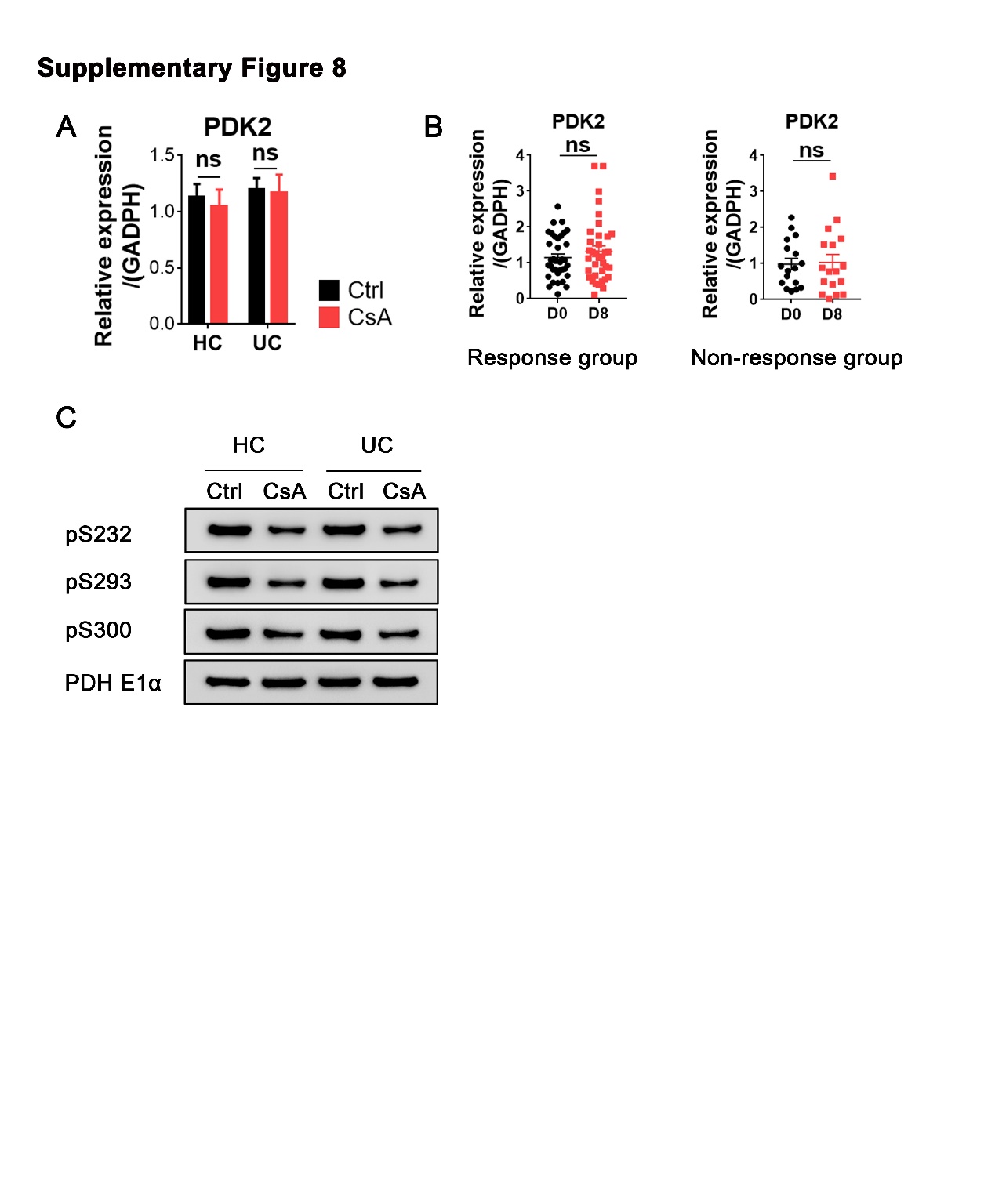
Fig. S8** CsA decreases the phosphorylation of the E1α subunit of PDH but does not influence on the expression of PDK2 in neutrophils. (A) Neutrophils (5 × 10^6^ cells) were isolated from ASUC patients (n = 27) and healthy donors (HC, n = 25) and cultured as described in Fig. 5A, and qRT-PCR was used to quantify the expression of PDK2. (B) Neutrophils (5 × 10^6^ cells) isolated from ASUC patients in the Response group (n = 36) and Non-response group (n = 17) before (D0) and on day 8 (D8) after treatment of CsA, and qRT-PCR was used to quantify the expression of PDK2. (C) Neutrophils (5 × 10^6^ cells) were isolated from ASUC patients and healthy donors, cultured as described in Fig. 5G, and WB was used to quantify the abundance and the phosphorylation of the E1α subunit of PDH. ns, not significant.

**
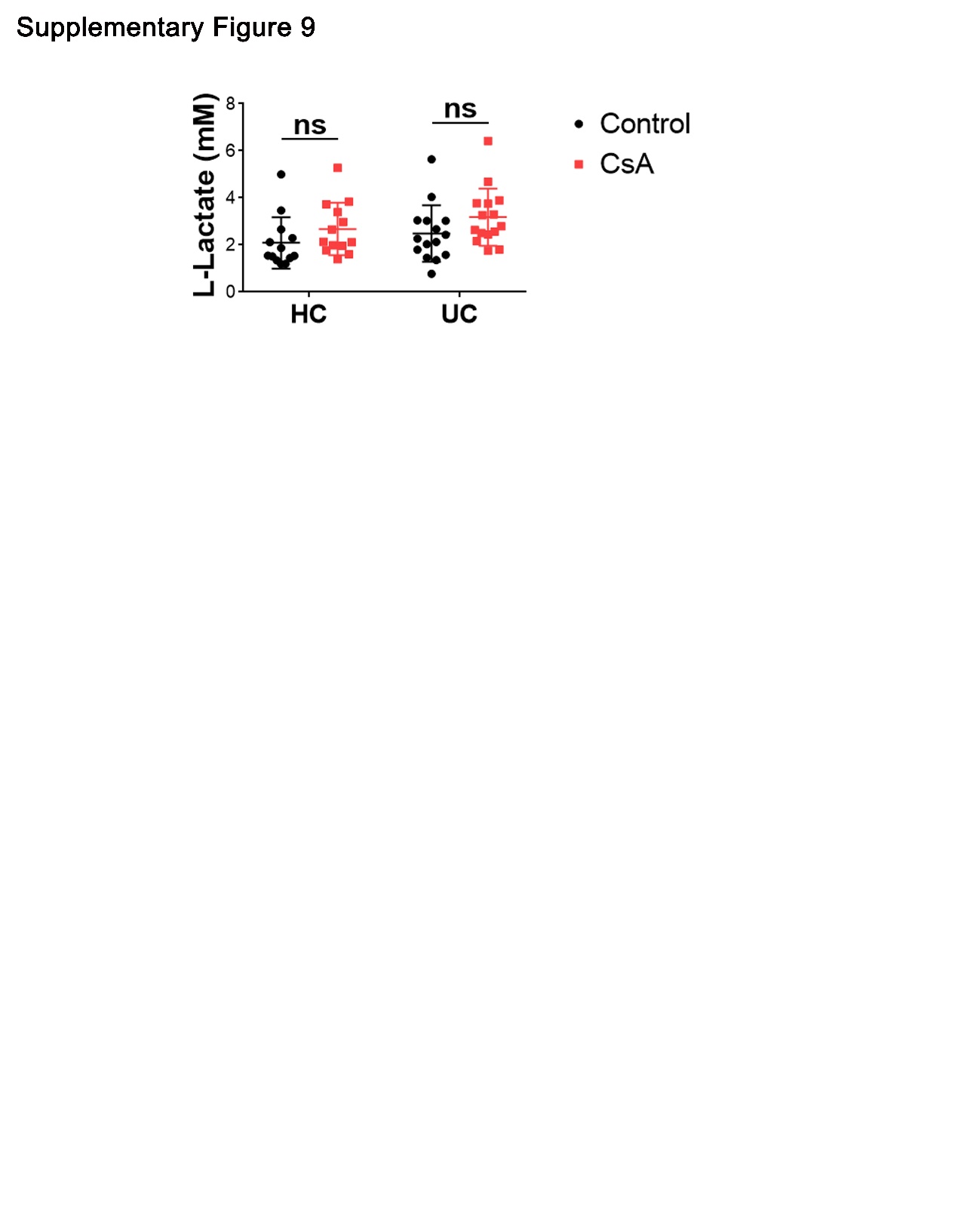
Fig. S9** CsA does not affect the expression of L-lactate. Quantitative analysis of L-lactate (HC, n = 13; UC, n = 15) was performed by Fluorometric Assay Kit. ns, not significant.

**
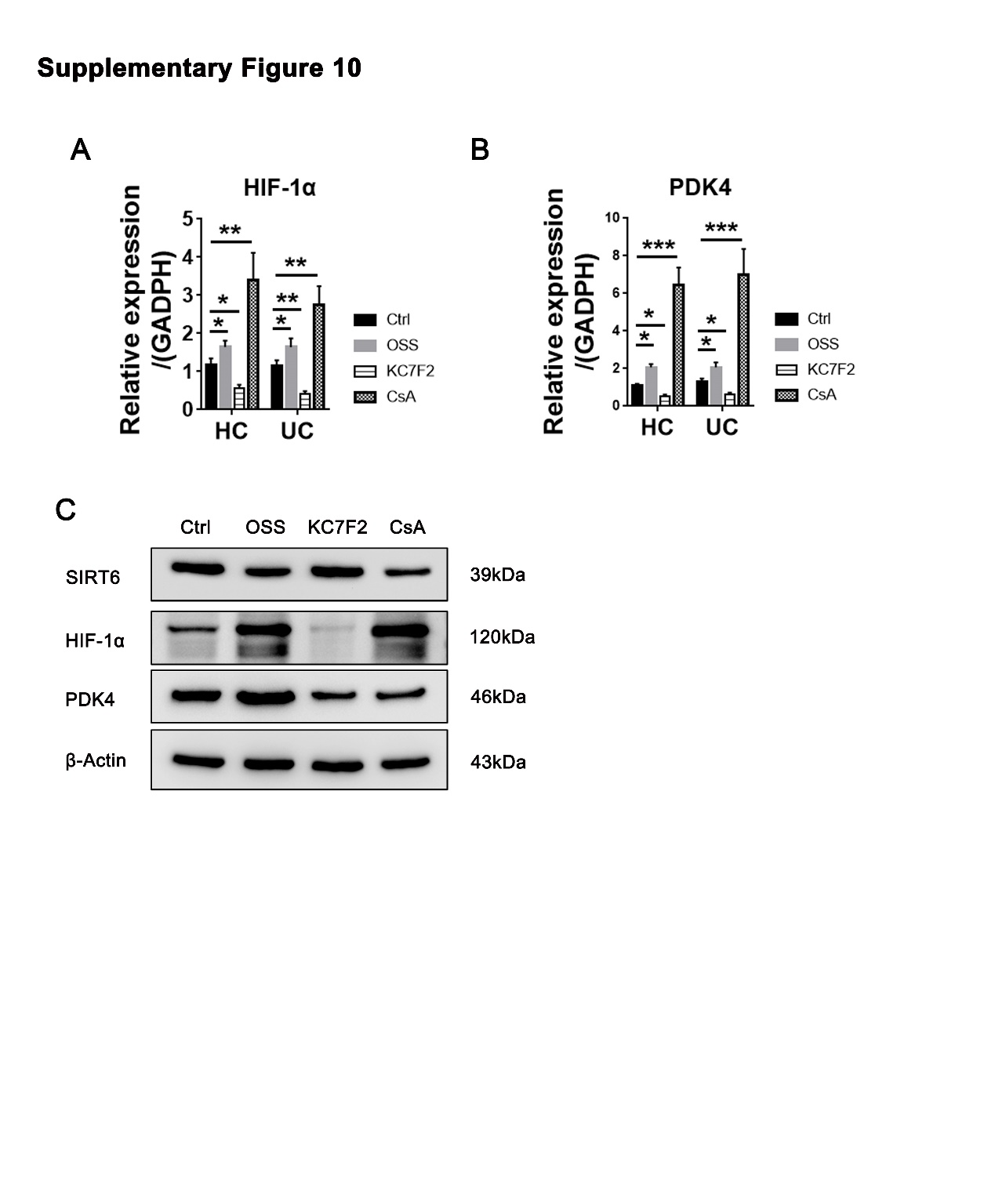
Fig. S10** Blockage of SIRT6 or HIF-1α regulates the expression of PDK4. (A-B) Neutrophils (5 × 10^6^ cells) isolated from peripheral blood of ASUC patients (n = 8) and healthy controls (HC, n = 10) were pretreated with OSS_128167 (an inhibitor of SIRT6, 100 μM/mL), KC7F2 (an inhibitor of HIF-1α, 20 μM/mL), and CsA (10 μg/mL), respectively, *in vitro* for 3 hours, and the mRNA levels of HIF-1α (A) and PDK4 (B) was determined by qRT-PCR. (C) Neutrophils (5 × 10^6^ cells/mL) isolated from ASUC patients were pretreated with OSS_128167 (an inhibitor of SIRT6, 100 μM/mL), KC7F2 (an inhibitor of HIF-1α, 20 μM/mL), and CsA (10 μg/mL), respectively, *in vitro* for 6 hours, and the level of SIRT6, HIF-1α and PDK4 were measured by WB. **P* < 0.05, ***P* < 0.01, and ****P* < 0.001.

**
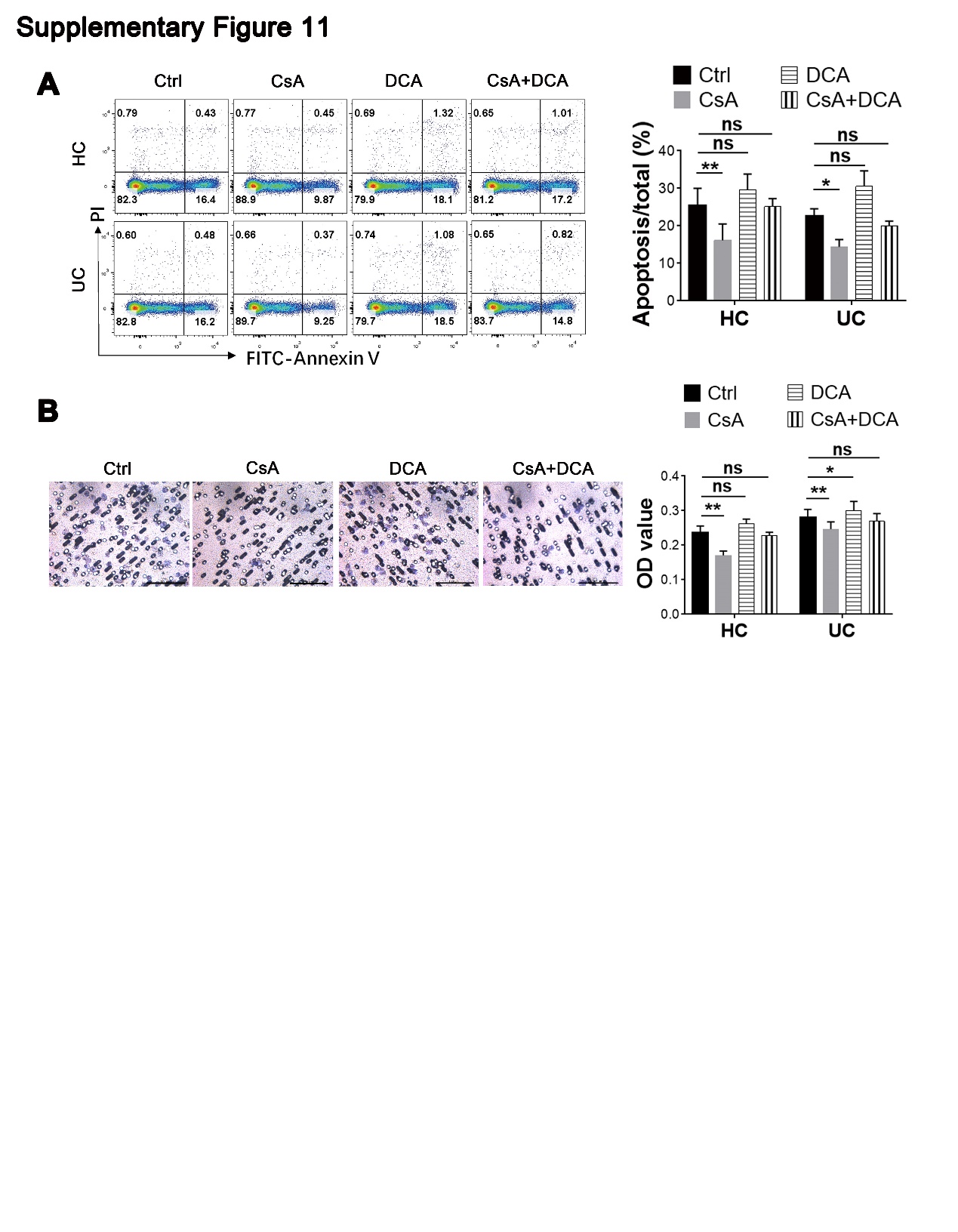
Fig. S11** Blockage of PDK4 regulates the function of neutrophils by inhibiting apoptosis and migration. (A-B) Neutrophils (5 × 10^6^ cells/mL) isolated from peripheral blood of ASUC patients (UC, n = 8) and healthy donors (HC, n = 10) were pretreated with DCA (an inhibitor of PDK4, 5 mM/mL) *in vitro* for 3 hours. (A) Representative images of apoptotic neutrophils stained with annexin V were measured by flow cytometry. The percentages of the apoptotic neutrophils were counted and shown in the right panel. (B) The migration of neutrophils was detected using Transwell chamber and stained with crystal violet, and the quantification of cell migration was shown in the right panel. Non-decolorized acetic acid was used as medium alone to reduce the impact of background. Scale bars: 100 μm. Statistical significance was assessed by Tukey’s test. Mean ± SEM are shown. **P* < 0.05 and ***P* < 0.01; ns, not significant.

**
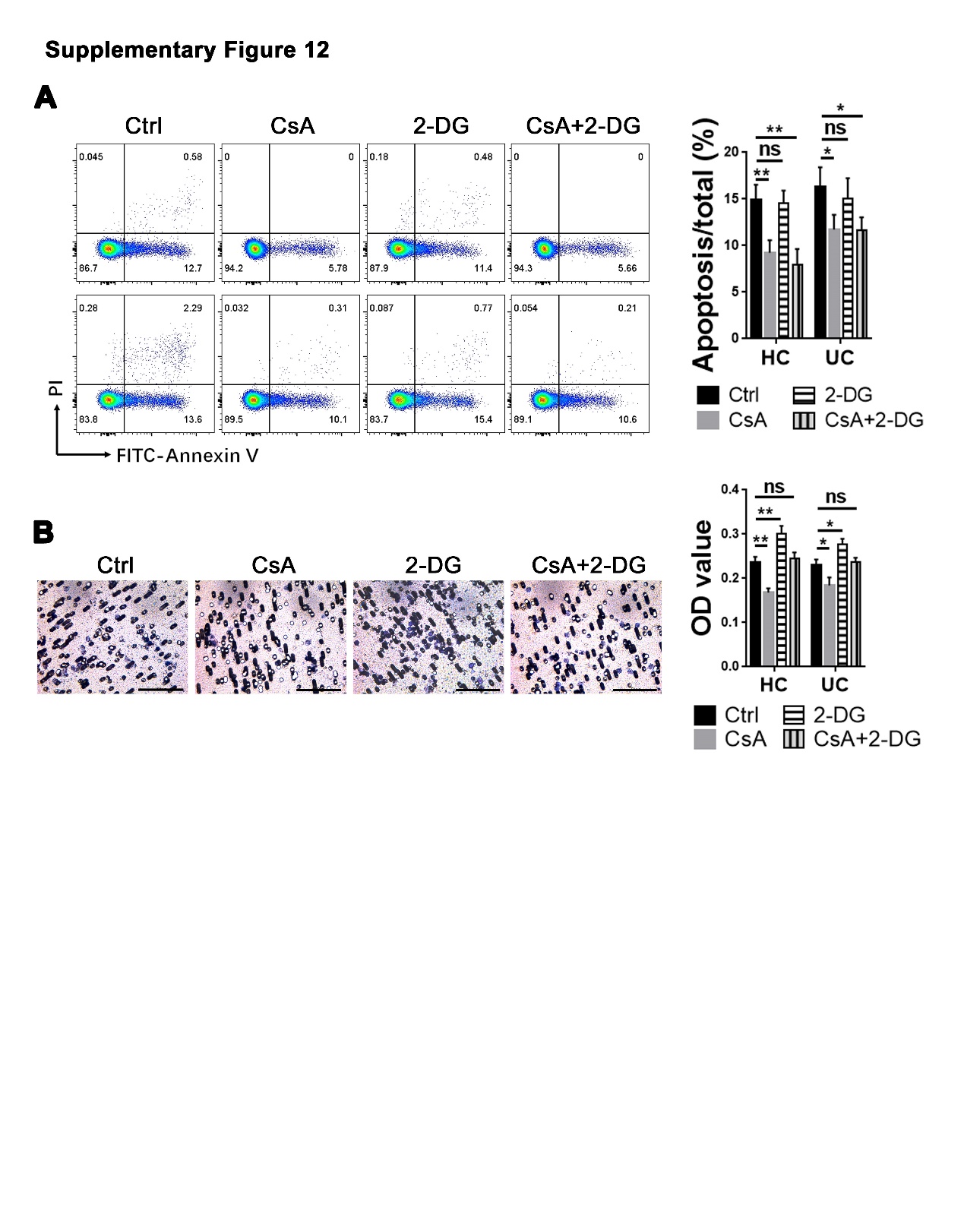
Fig. S12** Blockage of glycolysis regulates the function of neutrophils by inhibiting migration. (A-B) Neutrophils (5 × 10^6^ cells/mL) isolated from peripheral blood of ASUC patients (UC, n = 10) and healthy donors (HC, n = 10) were pretreated with 2-DG, an inhibitor of glycolysis (4 mM/mL), *in vitro* for 3 hours. (A) Representative images of apoptotic neutrophils stained with annexin V were measured by flow cytometry. The percentages of the apoptotic neutrophils were counted and shown in the right panel. (B) The migration of neutrophils was detected using Transwell chamber and stained with crystal violet, and the quantification of cell migration was shown in the right panel. Non-decolorized acetic acid was used as medium alone to reduce the impact of background. Scale bars: 100 μm. Statistical significance was assessed by Tukey’s test. Mean ± SEM are shown. **P* < 0.05 and ***P* < 0.01; ns, not significant.

**
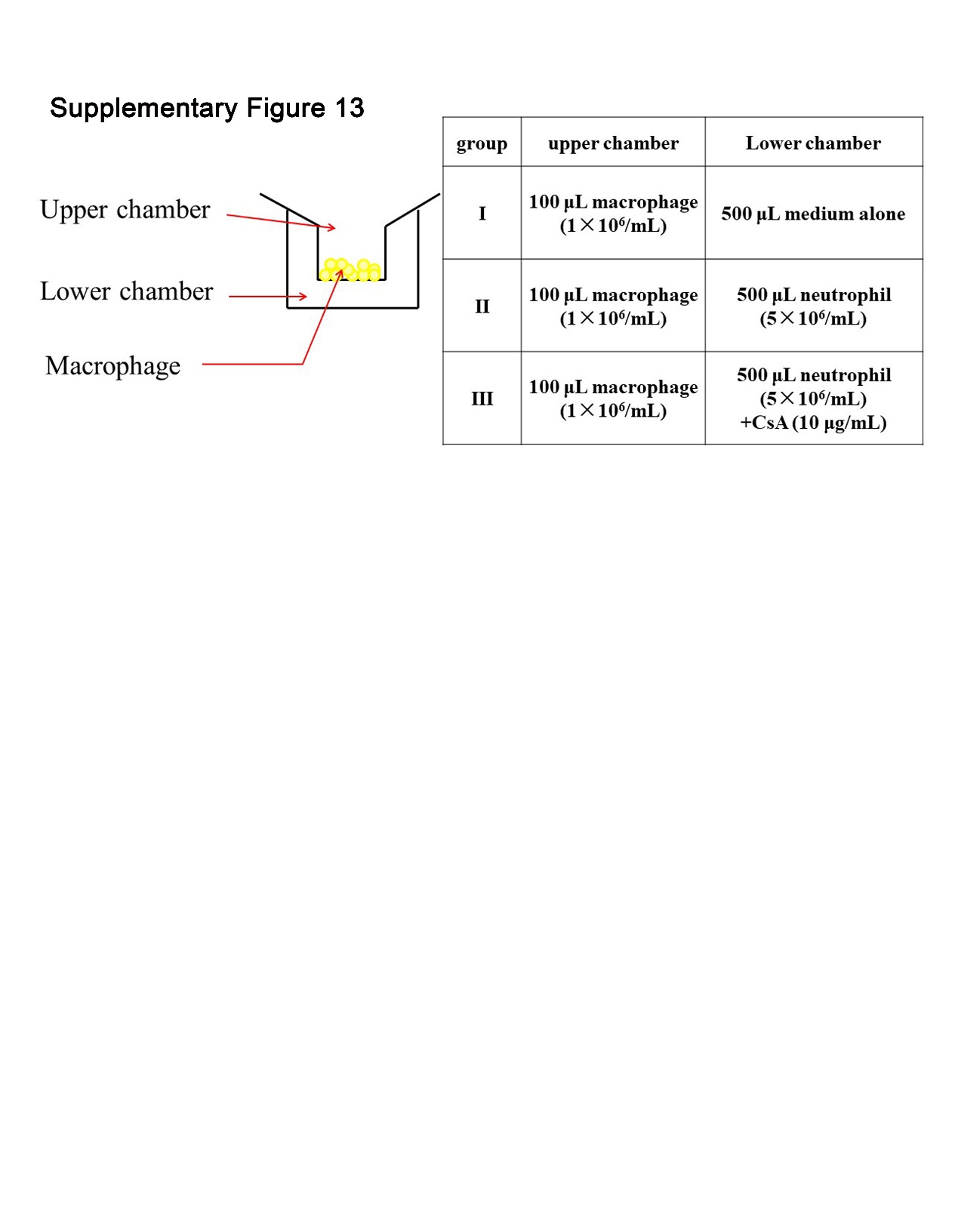
Fig. S13** Diagram of macrophage migration stimulated by neutrophils in the presence of CsA. Macrophages (1 × 10^5^ cells) isolated from mouse bone marrow were seeded in the upper well of Transwell chamber without serum. The bottom chambers were divided into three groups, in which culture medium alone without any cells or stimulants in the bottom chamber was set as group I (namely control group), the presence of neutrophils (2.5 × 10^6^ cells/mL) in the bottom chamber as group II, and the presence of neutrophils (2.5 × 10^6^ cells/mL) and CsA (10 μg/mL) in the bottom chamber as group III.

**Supplementary Methods**

**Fetal calprotectin assessment**

Stool samples were collected from ASUC patients (Response group, n=36; Non-response group, n=17) before and after CsA management, and analyzed by a Dry-Fluorescent-Immunoassay analyzer based on fluorescence immunochromatography assay (FICA), with an extended range from 15 to 2100 μg/g. The test was performed according to the manufacturer’s instructions (Guangzhou Forreal Biotech Co. Ltd; Guangzhou, China). In brief, samples were centrifuged after shaken into homogenate, and 90 μL supernatant was added to the sample well of the test card for 15 minutes before testing.

**Analysis of antimicrobial peptides and cytokines**

Neutrophils were incubated in medium alone or under stimulation with LPS (300 ng/mL), CsA (10 μg/mL), and LPS (300 ng/mL) together with CsA (10 μg/mL), respectively, for 3 hours and harvested, and total RNA was extracted using Trizol reagent (Life technologies). The quantity and quality of RNA were assessed on a Nanodrop2000 spectrophotometer (Thermo scientific; Waltham, MA, USA), with a 260/280 ratio of > 1.8 and 28S/18S ratio of >1.4 for the majority of the samples. mRNA reverse transcription was performed using a 5×All-In-One RT Master Mix kit (Applied Biological Materials Inc.; Richmond, BC, Canada). For mRNA expression analysis, qRT-PCR was performed using a SYBR Green PCR kit (TaKaRa; Dalian, China) according to the manufacturer’s instructions, and the mRNA levels were normalized to the expression of GAPDH. Each PCR amplification was performed in duplicate. Moreover, the supernatants were also collected for the detection of cytokines and antibacterial peptide using ELISA according to manufacturer’s instructions. The data were analyzed with Epoch Microplate Spectrophotometer (BioTek Instruments; Waltham, MA, USA), and the primers or reagents were listed in Table S2 or Table S3, respectively.

**Transwell chamber cell migration assay for macrophages**

Macrophages were resuspended in serum-free 1640 medium containing 1% P/S at 10^6^/mL. Thereafter, 100 μL of cells was seeded in the upper well of a Transwell chamber and 500 μL of 1% P/S serum-free 1640 medium was added into the bottom chamber. The assay was then divided into three groups. 2.5 × 10^6^ neutrophils were added into the bottom chambers in the absence or presence of CsA (10 μg/mL) as groups II and III, respectively, and medium alone in bottom chamber was set as group I (**Fig. S13**). After incubation for 12 hours, cells adherent to the membrane in a 3-μm pore Transwell system were fixed with 4% PFA for 10 minutes, stained with 1% crystal violet for 30 minutes, and then observed under a light microscope (×200).

**CD4^+^ T cell proliferation assay**

CD4^+^ T cells (5 × 10^5^ cells/mL) were cultured for 5 days and then stimulated with phorbol-12-myristate-13-acetate (PMA) (50 ng/mL; Sigma-Aldrich), ionomycin (1000 ng/mL), and golgi-stop (1 μg/mL; BD Biosciences), respectively, for 5 hours. After permeabilization with Fixation/Permeabilization Buffer Set ([Invitrogen](https://www.thermofisher.com/cn/zh/home/brands/invitrogen.html); Carlsbad, California, USA), cells were stained with fluorochrome-conjugated anti-IL-17A, anti-TNF-α and anti-IFN-γ antibodies, respectively. Expression of intracellular cytokines in CD4^+^ T cells was examined on a BD FACSCanto II (BD Biosciences; San Diego, CA, USA) and analyzed by FlowJoVX software (Tree Star, Inc.; Ashland, OR, USA).

**SIRT6 small interfering RNA (siRNA)**

siRNA transfection was performed according to the manufacturer’s instructions (Genepharma; Shanghai, China). Briefly, HL-60 cells (National Infrastructure of Cell Line Resource; Shanghai, China) were seeded at 2 × 10^5^ cells per well of 24-well plates. After 24 hours of culture, the cells were transfected with *SIRT6*-specific siRNA (sense 5`-3`: UCAUGACCCGGCUCAUGAATT; antisense 5`-3`: UUCAUGAGCCGGGUCAUGATT) or the scramble siRNA (50 nM) using lipofectamine 2000 (Invitrogen; Carlsbad, CA, USA) in 500 μL opti-MEM medium. The efficiency of knock-down was measured 48 hours post-transfection.

**RNA sequencing**

Total RNA was isolated from neutrophils with or without the pretreatment of CsA (10 μg/mL) using the protocol described above. The quantity and quality of RNA were assessed on a Nanodrop2000 spectrophotometer (Thermo scientific; Waltham, MA, USA). Total RNA was amplified and labeled by Low Input Quick Amp WT Labeling Kit (Cat. # 5190-2943, Agilent technologies; Santa Clara, CA, US), following the manufacturer’s instructions. Labeled cRNA was purified by RNeasy mini kit (Cat. # 74106, QIAGEN; Germany). Each slide was hybridized with 1.65 μg Cy3-labeled cRNA using Gene Expression Hybridization Kit (Cat. # 5188-5242, Agilent technologies) in Hybridization Oven (Cat. # G2545A, Agilent technologies), according to the manufacturer’s instructions. After 17 hours of hybridization, slides were washed in staining dishes (Cat. # 121, Thermo Shandon; Waltham, MA, US) with Gene Expression Wash Buffer Kit (Cat. # 5188-5327, Agilent technologies), followed by the manufacturer’s instructions. Slides were scanned by Agilent Microarray Scanner (Cat. #G2565CA, Agilent technologies) with default settings (Green Dye channel, 3 μm Scan resolution, 100% PMT, 20bit). Data were extracted with Feature Extraction software 10.7 (Agilent technologies). Raw data were normalized by Quantile algorithm, and limma packages in R. Gene ontology analysis was used for annotation, visualization, and Integrated Discovery (DAVID) bioinformatics. Sequencing libraries were generated using NEBNext® Ultra^TM^ RNA Library Prep Kit for Illumina® (NEB; San Diego, CA, USA).

**Western blotting analysis**

With the exception of immunoblotting for Akt, HIF-1α, PDK4 and SIRT6, neutrophils were first incubated with CsA (10 μg/mL) for 6 hours, and then lysed in RIPA buffer supplemented with protease inhibitors. Lysates mixed with SDS sample buffer were boiled and resolved by SDS-PAGE. Bandings were transferred to PVDF Immobilon P membranes using a wet transfer, blocked by TBST containing 5% non-fat milk, and incubated overnight at 4˚C with primary antibody in the manufacturer-recommended staining solution. Details of antibodies were shown in Table S3. Immunoblots were visualized using the Odyssey West ECL system (Lincoln, NE, USA) followed by film exposure. Quantification of digitally scanned immunoblots was performed by densitometry analysis using ImageJ software.

**PDK4 ubiquitination**

To detect PDK4 ubiquitination in human neutrophils, cells were stimulated with or without CsA (10 μg/mL) for 6 hours and subsequently lysed in RIPA buffer. The cell lysate was incubated with anti-PDK4 and protein-G agarose beads overnight at 4°C. The beads were extensively washed with the RIPA buffer four times. Finally, the lysate was boiled for 10 minutes with SDS loading buffer.

**Immunohistochemistry staining**

To localize neutrophil, CD4^+^ T cell and macrophage infiltration in colon mucosa from UC patients before and after treatment of CsA, colon biopsies were fixed in 10% paraformaldehyde and embedded in paraffin. 5-μm-thick sections of colon tissues were cut and stained immunohistochemically. Antigen retrieval was performed using boiled ethylenediaminetetraacetic acid-Tris buffer (pH 9.0), and the sections were then treated with 3% H_2_O_2_ at 37°C for 15 minutes, followed by blocking with goat serum for 30 minutes, and stained with anti-MPO mAb (1:100; Abcam), anti-CD4 mAb (1:500; Abcam), and anti-CD68 mAb (1:100; Abcam), respectively, as primary antibody at 4°C overnight. After washed 3 times with TBS, the sections were incubated with HRP-conjugated goat anti-IgG at room temperature for 1 hours, followed by treating with 3,3′-diaminobenzidine for color reaction and then counterstained with hematoxylin.
